# SCPA-Net: Text-Enhanced Cross-Platform Framework with Semantic Consistency Enhancement for Pine Wilt Detection

**DOI:** 10.3390/plants15111744

**Published:** 2026-06-04

**Authors:** Shicong He, Weizhi Zhao, Peng Wang, Mingfang He

**Affiliations:** School of Electronics, Information and Physics, Central South University of Forestry and Technology, Changsha 410004, China; heshicong88@gmail.com (S.H.); t20162306@csuft.edu.cn (M.H.)

**Keywords:** pine wilt disease, UAV remote sensing, satellite remote sensing, cross-domain remote sensing, text-based multimodality

## Abstract

With the rapid development of UAV and satellite remote sensing, in combination with deep learning, high-efficiency monitoring of pine wilt disease (PWD) for forest health management is now feasible. Accurate detection has not yet been realised. The sensing platforms have different ranges of space, observation areas and imaging orientations. At the same time, the target groups for PWD often have weak phenotypic features, are easily affected by a complex forest background, and show irregular data distributions at different stages of the disease. The above factors are limits to the performance of traditional methods based only on general visual features. To address the problems mentioned above, we propose the cross-platform semantic-consistent and phenotype-adaptive detection network SCPA-Net for high-precision PWD detection in both UAV and satellite images. First, we construct a cross-platform multimodal framework to integrate remote sensing images and disease-related text descriptions. The above design adds semantic prior knowledge to expand the model’s capacity for high-level phenotypic attribute extraction without direct observation. Second, to reduce the semantic gap caused by the different platforms, improve the semantic consistency of UAV and satellite images, strengthen discriminative feature channels and salient regions, and address cross-platform misalignment. Third, since the targets are often associated with complex forest environments, target-context relational modeling is enhanced and irrelevant interference is suppressed to reduce the impact of non-causal attributes. As pine wilt disease symptoms gradually progress from mild to severe (e.g., crown discoloration, texture variation, and wilting severity), differences among disease stages may lead to learning imbalance and knowledge forgetting; therefore, a staged adaptation strategy has been proposed. First, the model learns from relatively easy examples. Subsequently, it progressively learns from more difficult examples to enhance generalization performance. Experiments have been conducted on a self-built cross-platform dataset, a satellite dataset, the PDT public dataset, and the Roboflow dataset, and the proposed method has achieved better detection accuracy and generalization. The framework can address the problem of PWD detection in challenging-to-process forestry remote sensing data reasonably well.

## 1. Introduction

Pine wilt disease (PWD) is a serious forest disease that threatens the resources of pine forests around the world. China, Russia and the United States are some of the main production areas for pine trees [1]. It is spreading rapidly, has a high death rate, and will soon be spread widely. The pathogen spreads quickly through the phloem and xylem of trees to cause a widespread decline in pine trees. Therefore, there will be considerable economic losses in forestry and instability of the forest ecosystem. At the same time, the old chemical control method may leave pesticide residues and cause soil acidification. These limitations are not conducive to sustainable forest management or precise disease control. Therefore, efficient and accurate detection methods for PWD need to be developed. Remote Sensing can also be applied to address this problem in practice. UAVs and satellites can be used to conduct rapid, automatic, large-scale detection of diseased trees. The function above offers technical support for early warning, accurate control, reduction of economic loss and protection of the ecological environment [2].

Field observations are commonly conducted to investigate disease conditions and environmental characteristics of trees. However, these approaches are labour-intensive, time-consuming, and limited in spatial coverage. The above deficiencies are not suitable for large-scale, dynamic forest disease monitoring [3]. Traditional methods of image processing are used for threshold segmentation, edge detection and texture analysis in feature extraction from diseased tree crowns. The working conditions in a dense forest are generally not favorable. It is even more severe in the presence of background interference, canopy occlusion and a weak phenotypic expression in the early stages of disease. Remote sensing is an effective approach for large-scale and time-series observations, supporting forest disease surveys. However, these approaches are still limited by spatial resolution, imaging scale, and scene complexity. As a result, the initial indicators and particular targets of the disease in PWD remain unknown. Machine learning is now used to improve the accuracy and efficiency of detection for remote-sensing-based PWD detection. The above ways learn discriminative features from labelled samples and improve the performance of classification and prediction. Most of the old machine learning methods are still based on manual feature engineering and do not handle complex cases well. Deep learning can automatically learn multi-level feature maps from large-scale remote sensing images at the same time. Therefore, they are more suitable for all kinds of forest conditions and complex backgrounds, providing a relatively practical solution for high-speed, high-accuracy, and automated PWD detection.

Recently, many studies have been carried out on the remote sensing detection of pine wilt disease (PWD). Du et al. proposed an improved YOLOv5L-s-SimAM-ASFF model for diseased tree detection in UAV RGB imagery [4]. Their method achieved high detection accuracy and efficiency for UAV-based pine wilt disease monitoring. However, it mainly relies on single-platform visual information and lacks cross-platform modeling capability. In addition, Su et al. extended PWD-YOLOv8 for UAV imagery by incorporating attention mechanisms and feature enhancement strategies to improve detection performance under complex environmental conditions [5]. Although the above improvements have been made, they are still single-modal and do not fully exploit the semantic information of disease phenotypes. Wu et al. applied deep object detection models to UAV remote sensing data, demonstrating that deep learning can improve the localization accuracy of diseased regions [6]. The above way is also not suitable for a complex forest environment or a small target. Liu et al. employed UAV RGB imagery and hyperspectral reconstruction to build an early warning system for PWD using SVM. These studies demonstrated that multi-source data can improve the early detection of pine wilt disease by enhancing the identification of subtle disease symptoms [7]. Their basic design is for classification, and they are not directly suitable for high-efficiency object detection frameworks. Wang et al. have applied high-resolution satellite imagery and semi-supervised deep learning to detect discoloured trees caused by PWD in a satellite-based system [8]. Lv et al. have added a self-attention mechanism to improve the global feature model in the analysis of high-resolution satellite images [9]. Under the circumstances of satellite imaging, detailed disease phenotypes are often lost. Many of the previous studies have not presented an integrated model for UAV and satellite image analysis. Although the above methods have shown some results in the detection of single-platform PWD, they are not ideal. They include the absence of explicit modelling for disease-related colour and phenotypic priors, insufficient semantic consistency between UAV and satellite platforms, and unstable performance in the presence of weak phenotypic indicators and noisy backgrounds [10].

Although there has been some progress in remote sensing-based recognition and detection of pine wilt disease (PWD) in previous studies, three major deficiencies remain for cross-platform detection scenarios.

**Figure 1:**
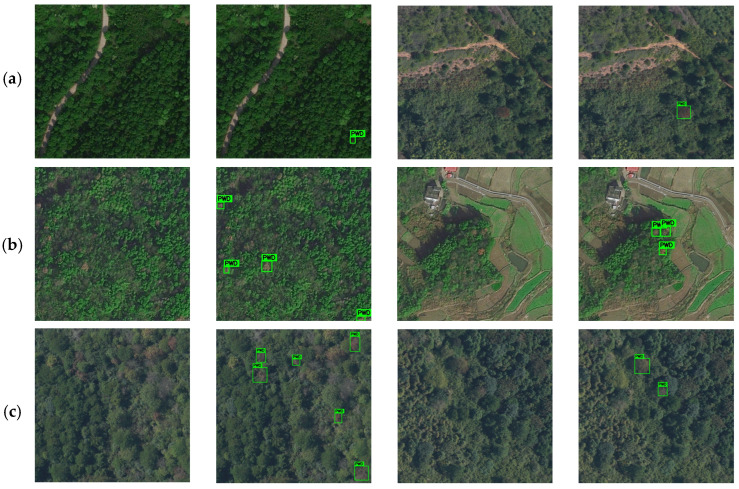
Representative challenges for pine wilt disease (PWD) target detection under cross-platform remote sensing scenarios. (**a**–**c**) show representative examples of different detection challenges discussed in the Introduction (Ground truth images manually labelled next to the original image).

(1) As shown in Figure 1a, Satellite and UAV remote-sensing images have different spatial resolutions, observation scales, imaging perspectives and texture details. Therefore, due to the above reasons, the feature distribution and high-level semantic representation of PWD tree crowns vary among platforms. Most of the existing methods have been developed for a single platform and do not have good constraints on high-level semantic consistency between UAV and satellite images. As a result, there is a risk of feature misalignment and performance degradation in cross-platform transfer and joint detection.
(2) As shown in Figure 1b, PWD tree crowns in a complex forest environment are exposed to shading caused by shadows, bare ground, healthy crowns and other areas with similar textures. In such cases, the background context frequently occurs simultaneously with the diseased target, and the model may learn spurious correlations as a result to produce false alarms. Existing detection methods are generally local convolution-based representations or simple attention augmentations. However, they do not explicitly model the heterogeneous relationships among the target areas and background areas, and they lack a causal mechanism for suppressing background confounding effects. Therefore, the current models are still unable to recognise the actual diseased area in a cluttered background consistently.
(3) As shown in Figure 1c, the PWD tree crowns have shown various phenotypes at different stages of the disease. Diseased trees in their early stages generally have only a mild change in colour, blurred boundaries and weak reactions, so they are much harder to detect than those in the middle and later stages. Most of the current methods are single-stage static training models that mix samples from all stages and learn them at the same time. Such a strategy does not have a continuous learning mechanism to keep up with the progress of weak phenotype cognition. Therefore, when the model needs to adapt to early-stage ambiguous diseased trees, it is often dominated by more obvious late-stage samples. At the same time, cross-stage learning may result in forgetting the previously learned knowledge. It will reduce the detection rate of early weak-phenotype trees and generally weaken the model.

To strengthen the representation and transferability of high-level semantics for PWD tree crowns in UAV/Satellite cross-platform scenarios, recent research has begun to explore cross-platform detection, cross-domain adaptation, and semantic alignment of multi-source remote sensing images. Cai et al. have used UAV RGB images and Sentinel-2 satellite multispectral data together for individual tree disease diagnosis, and have shown that combined data from both platforms can provide full support for diseased target recognition; thus, it is proposed that integrated remote sensing observations be carried out in disease monitoring [11]. Wang et al. have utilised GF-2 satellite images to identify colour changes in trees caused by PWD in a semi-supervised deep learning method, and have shown that remote sensing data can be used to monitor the spread of this disease on a large scale [8]. At the same time, Ma et al. proposed a hierarchical alignment network for remote sensing object detection and cross-domain modeling. Their method addresses cross-domain shifts in aerial imagery through shallow image-level alignment and deep instance-level alignment. The results demonstrated that semantic consistency constraints can improve domain transfer performance in remote sensing tasks [12]. Qi et al. have also added modality alignment and feature fusion mechanisms to cross-modal remote sensing object detection, improved the collaborative representation ability of different modalities through spatial alignment and channel consistency optimisation, and shown that explicit alignment of high-level semantic relationships can enhance the robustness of object detection in complex remote sensing environments [13]. Although the above studies have improved the target recognition performance in remote sensing to a certain extent, most of the existing methods are still mainly focused on single-platform modeling, or only perform general alignment for cross-domain/cross-modal differences, and lack dedicated modeling for the inconsistency of high-level semantics of PWD tree crowns in UAV/Satellite cross-platform scenarios. At the same time, most of the above methods still mainly use implicit adaptation of networks to address cross-platform differences and lack a mechanism that jointly integrates high-level semantic alignment with spatial-channel dual-attention enhancement. Therefore, due to the complexity of the background, weak phenotypic features, and joint detection scenarios across multiple platforms, the representation of high-level semantics for diseased objects is still lacking.

To reduce the problem of false detection caused by complex backgrounds and contextual co-occurrence or spurious correlation factors in remote sensing, recent research has begun to explore the application of graph-based relational modeling, causal learning and interventional inference for target recognition in remote sensing. Tian et al. have proposed a relation-augmented embedded graph attention network to improve the feature representation ability of remote sensing object detection by explicitly modeling relational dependencies among targets, and have shown that graph-based models can be applied to capture target association information in complex remote sensing scenarios [14]. Kaur et al. have also studied the application of multimodal graph neural networks in Earth observation and believe that, due to the heterogeneity of graph representations, more nodes, relationships and other context information can be incorporated to improve the robustness of representations under complex remote sensing conditions [15]. At the same time, Zhang et al. have put forward a causal learning mechanism for multimodal remote sensing classification tasks. By suppressing non-causal interference factors, the method enhances the capacity of the model to extract genuine discriminative information, and thus causal modelling can reduce the influence of spurious correlations in remote sensing applications [16]. Akçay et al. have shown that by combining graph neural networks with causal machine learning, graph-structured dependency models and causal effect analyses can be used to find more stable and reliable factors in complex relationships [17]. Although the above studies have improved recognition performance in complex remote sensing situations to some extent, most of the existing methods are mainly for general remote sensing classification, detection or environmental prediction tasks, and lack specific models that consider the heterogeneous relationships among pine wilt disease tree crown targets and background contexts. In addition, there are still no mechanisms to combine various graph neural networks with interventional causal inference jointly. Therefore, in a complex forest environment, the targets of pine wilt disease are still easily affected by shadows, bare land, healthy tree crowns and other regions with similar textures; thus, spurious correlation expressions induced by background co-occurrence occur, and the stable discriminative capability of the model for diseased targets is reduced.

To improve the stability and robustness of pine wilt disease (PWD) tree crown detection in the face of various sample conditions at different times, some research has started to explore how continual learning, incremental learning and anti-forgetting mechanisms can be applied to remote sensing vision tasks. Li et al. have built a continuous learning benchmark for remote sensing scene classification (CLRS) and pointed out that as remote sensing data accumulates and tasks are expanded, deep models tend to suffer from catastrophic forgetting after learning new data; thus, continual learning is needed to extend the application scope of deep learning in remote sensing scene understanding [18]. Peng et al. have also put forward a continuous contrastive learning method for cross-dataset remote sensing scene classification that mitigates the forgetting of old knowledge by improving feature discriminability during the continual learning process, and have shown that continual learning mechanisms can enhance the cross-stage adaptation ability of remote sensing tasks [19]. Feng et al. have been studying how to deal with the problem of class-incremental learning in remote sensing object detection, observing that when training only with new data, the previously learned knowledge may be lost rapidly; therefore, experience replay and other knowledge-retention mechanisms need to be used to reduce catastrophic forgetting in these remote sensing detection tasks [20]. Some research has also reduced the damage caused by new task learning to old task representations in the context of few-shot incremental learning and resolution-incremental learning by employing methods such as memory replay, knowledge distillation, prototype calibration and parameter regularization to improve continual adaptation ability and generalisation performance. Although the above studies have demonstrated the effectiveness of continual learning for knowledge retention in remote sensing tasks to some extent, most of the existing methods are mainly designed for general remote sensing scene classification, class-incremental recognition, or resolution transfer problems, and lack specific designs for the progressive evolution process of disease phenotypes from obvious to ambiguous in PWD detection scenarios. In the early stages of the disease and under weak phenotypic conditions, the samples collected at different times show relatively large differences in the prominence of phenotype, border distinctness and differentiation difficulty. Existing methods cannot construct a progressive learning task flow in line with the evolutionary pattern of phenotypes, and they are unable to maintain a stable memory of previously learned mid- and late-stage disease knowledge when adapting to new weak-phenotype samples. As a result, there will be some loss of knowledge and performance.

To address the problems of high-level semantic inconsistency, spurious correlation interference from complex backgrounds, and difficulty in continual adaptation to weak-phenotype samples in cross-platform pine wilt disease (PWD) target detection, this paper introduces a new cross-platform semantic consistency and phenotype adaptation detection network called SCPA-Net. The new network takes remote sensing images and disease description text as inputs, and based on three main directions, has been developed: cross-platform semantic consistency enhancement, robust relational modelling in complex backgrounds, and progressive adaptation to weak-phenotype samples. The goal of this study is to develop a cross-platform remote sensing detection framework for PWD that can improve semantic consistency across UAV and satellite platforms, reduce spurious correlation interference from complex forest environments, and enhance continual adaptation to disease progression stages, thereby improving detection accuracy and cross-platform generalization capability. Specifically, this study focuses on three tasks: constructing cross-platform semantic consistency modelling mechanisms, strengthening robust disease-background relational representation, and improving progressive adaptation to weak-phenotype disease samples. This design will improve the detection accuracy of PWD targets in the model, enhance cross-platform compatibility, and strengthen the stability of detection under adverse conditions. The primary contributions of this paper are as follows.

(1) To deal with the problem of high-level semantic shift caused by different resolutions, scales and viewpoints of UAV and satellite remote sensing images, a Cross-Platform Semantic Consistency Enhancement (SCE) module is proposed. Increase the consistency of high-level semantic representations for diseased targets across platforms and thus reduce cross-platform semantic misalignment in this module. Enhance the feature responses of the discriminative channels and salient regions to improve the cross-platform recognition capability of the model for pine wilt disease targets.
(2) To solve the problem of easy interference caused by the background in complex forest scenes for diseased targets and to avoid spuriously correlated responses, a Spurious-Correlation Suppression Relational Modeling module (SSRM) is proposed. Strengthen the relational modelling capability of target regions and background regions in this module, and reduce non-causal interference from a complex background. Therefore, the model will be more suitable for extracting actual disease-related discriminative features and improving the detection accuracy and stability in complex situations.
(3) To address the problem of knowledge forgetting and performance fluctuations in the progressive transition of pine wilt disease phenotypes from obvious to ambiguous, a Progressive Phenotype Adaptation Mechanism (PPAM) has been proposed. In this way, a progressive phenotype task sequence from easy to difficult can be created to improve the continuous adaptation ability of the model to samples at all stages of the disease. Jointly optimise the retention of old knowledge and the acquisition of new knowledge to improve learning stability for weak-phenotype samples and cross-stage complex samples effectively.
(4) A cross-platform semantic consistency and phenotype adaptation detection network named SCPA-Net has been built for pine wilt disease cross-platform detection. A network of multiple data types has been built, and these will be combined in a three-part collaborative optimisation framework for SCE, SSRM and PPAM. Based on the results of the experimental tests, the proposed network has shown good performance in module effectiveness, comparison tests and generalisation tests. Based on the above results, this method can stably improve the cross-platform semantic consistency model, suppress interference caused by complex backgrounds, and enhance adaptation to weak-phenotype samples; thus, both the overall detection accuracy and robustness, as well as the cross-platform generalisation ability of the model, will be improved.

## 2. Materials and Methods

### 2.1. Dataset Acquisition and Processing

A self-built cross-platform remote sensing dataset was constructed for pine wilt disease (PWD) target recognition in this study. The dataset includes both UAV and satellite remote sensing images and supports cross-platform model training and validation. The training set contains 750 UAV images and 750 satellite images, while the validation set contains 200 satellite images. Satellite imagery was exclusively used for validation because the primary objective was to evaluate the model’s cross-platform generalization capability under satellite conditions. Compared with UAV imagery, satellite imagery presents greater challenges due to lower spatial resolution, less distinguishable disease symptoms, and more complex scene backgrounds. Therefore, satellite-based validation provides a more rigorous assessment of model generalization performance. By jointly utilizing UAV and satellite data, the constructed dataset integrates fine-grained disease information from high-resolution UAV imagery and large-scale target distribution information from satellite imagery, thereby providing a comprehensive data foundation for cross-platform disease target modeling.

In 2024, the UAV data used in this study were provided by the Hunan Academy of Forestry, China. Our research group was responsible for data preprocessing and subsequent dataset construction. A Zhongheng CW-15 unmanned aerial vehicle data acquisition platform with a flight altitude of more than 500 m was employed. A full-frame camera and a 35 mm lens with a 61-megapixel sensor were used for UAV image acquisition. The Ground Sampling Distance (GSD) of the resulting imagery is about 10 cm. The area of the forest in the UAV images is about 1174.5 km^2^, and it is a large-scale and complex scene. Given the large coverage of each high-altitude UAV image, there is generally a considerable amount of redundant background information in the original images. Therefore, the original large-scale images were first cropped, and then only the effective image samples containing PWD targets were selected for the construction of the dataset.

The satellite data come from the Beijing-3 satellite, and the coordinate system is WGS84. The areas for data collection are generally in several representative areas of Hunan Province, such as the vicinity of Dingcheng District in Changde City, Yongding District and Cili County in Zhangjiajie City, Shaoyang County in Shaoyang City, and the southern part of Shaoyang County near the border with Dong’an County in Yongzhou City. These areas have different terrains, such as plains, hills and mountains, and are therefore relatively complex in the background and varied in phenotype. Similar to the UAV images, the original satellite images were also cropped first, and then only the effective samples containing PWD targets were selected. Thus, the spatial context information of the areas is preserved, but the proportion of effective disease targets in the samples has been increased to improve the suitability of the dataset for the next stage of model training and evaluation.

All images were first converted into JPG format and annotated using the LabelMe tool. Forestry specialists manually labeled the pine wilt disease (PWD) target regions, and the annotation information was stored in JSON format. To reduce the risk of overfitting and improve training efficiency, all images were uniformly resized to 640 × 640 pixels before being fed into the network. The annotation results were subsequently used to generate supervision information for model training and validation.

The two types of data in the dataset are high-resolution disease symptom data collected by UAVs and large-area observations with more complex background environments obtained through satellites. Combine the two types of imagery to build a self-made dataset that can comprehensively cover the remote-sensing features of PWD targets at various scales and resolutions in different scenes. In short, the cross-platform dataset we have built here integrates the high-resolution detail of UAV imagery with the large-scale scene coverage of satellite imagery to provide a stable foundation for model training and validation in this paper.

In addition to image preprocessing, disease-related text descriptions were constructed to provide semantic information for multimodal learning. The text descriptions were manually generated according to observable pine wilt disease (PWD) characteristics, including crown discoloration, texture variation, wilting severity, and boundary distinctness. Specifically, text generation followed a rule-based template strategy, where disease-related attributes were combined according to visual observations and disease progression stages. Representative descriptions include “slight yellowing crowns with blurred boundaries and subtle texture changes” for early-stage disease, “reddish-brown crown discoloration with expanded affected regions” for middle-stage disease, and “gray-white wilted crowns with severe structural degradation” for late-stage disease. Each image sample was assigned a corresponding disease description according to its annotated disease characteristics. The text construction process followed disease interpretation criteria adopted during dataset annotation and was reviewed by forestry specialists involved in image labeling to ensure semantic consistency and biological relevance.

#### Feature Analysis of Samples at Different Phenotypic Stages of Pine Wilt Disease

**Figure 2:**
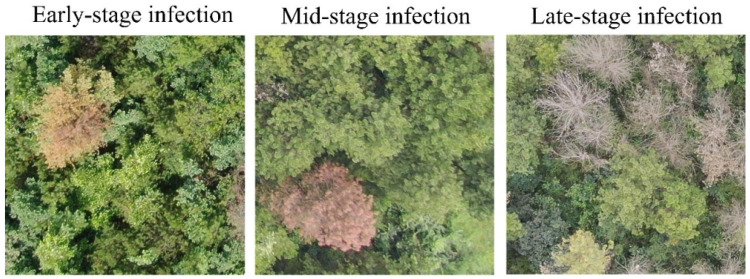
Pine Infection at Various Stages.

As shown in Figure 2, Pine wilt disease (PWD) manifests differently at different times in a tree’s life. At the beginning of the infection, the diseased crown of the tree is usually only slightly yellow or locally chlorotic, and the change in colour is relatively small. The damaged region and the adjacent healthy region at the crown are not clearly separated. In addition, they are easily influenced by light conditions, have similar canopy textures, and are prone to interference from background vegetation, thus posing a high recognition difficulty. In the middle stage of infection, there is more colour loss in the crown of the diseased tree. They generally have a relatively distinct yellow-brown or reddish-brown colour, and the area of damage spreads later in development. Increase the Distance of the target region from the background. But they are not rigid lines. Thus, the time before this can be viewed as when the weak phenotypes gradually turned into strong phenotypes. The crown of the diseased tree generally shows widespread dieback in the late stage of infection. They are generally light greyish-white or pale brown in colour and wilt easily. Colour and Texture Features are not the same as those in the nearby healthy vegetation. Therefore, the targets are relatively clear and thus have a lower detection difficulty.

To illustrate the construction of disease-related semantic descriptions, representative examples corresponding to the images shown in Figure 2 are provided. For the representative early-stage infection image, the semantic description is: “The pine crown exhibits slight yellowing and local chlorosis with blurred boundaries. The diseased region shows weak texture variation and remains highly similar to surrounding healthy vegetation.” For the representative middle-stage infection image, the semantic description is: “The diseased crown presents obvious yellow-brown discoloration with expanded affected regions and increased texture differences from surrounding vegetation.” For the representative late-stage infection image, the semantic description is: “The crown exhibits widespread dieback with gray-white appearance and clear structural degradation, showing obvious differences from surrounding healthy vegetation.” These descriptions are only representative examples corresponding to the images shown in Figure 2 and are provided to demonstrate the construction process of disease-related semantic texts.

### 2.2. SCPA-Net

**Figure 3:**
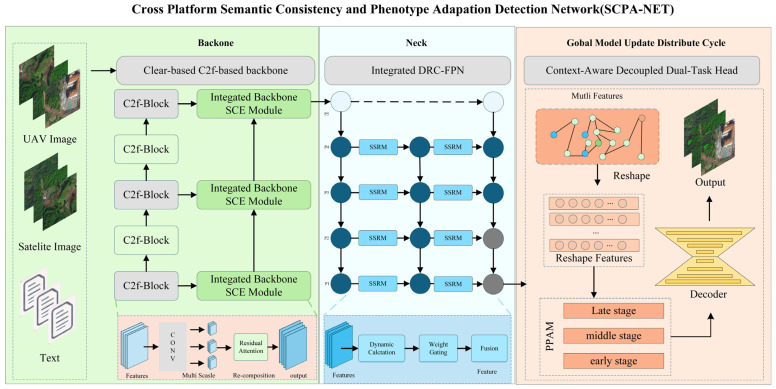
Structure of SCPA-Net.

**Figure 4:**
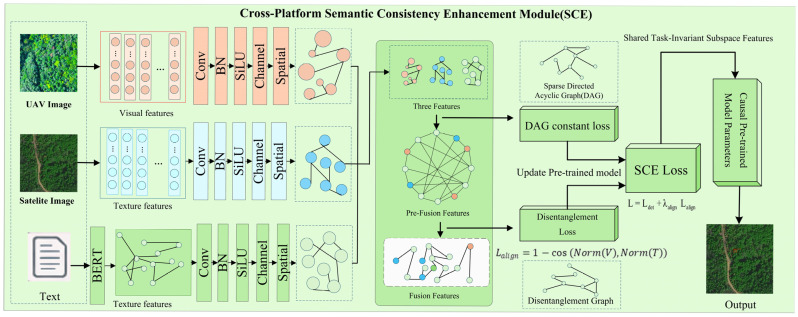
Structure of SCE.

**Figure 5:**
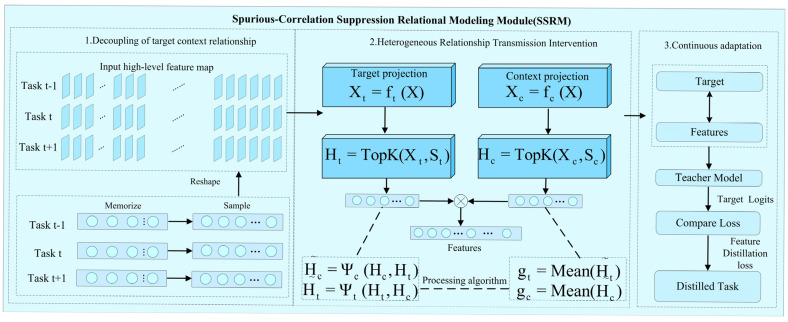
Structure of SSRM.

**Figure 6:**
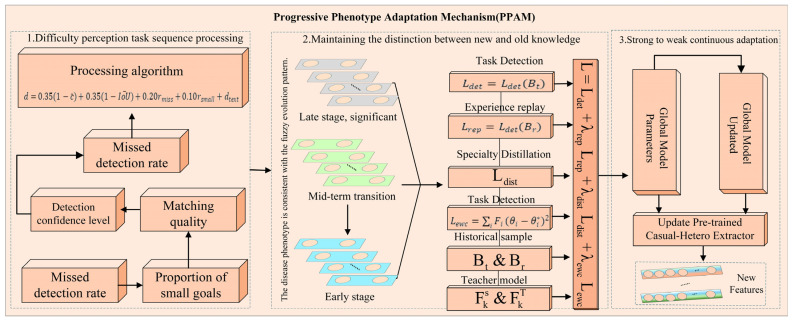
Structure of PPAM.

SCPA-Net is a new YOLOv10-based network. First, UAV and satellite remote sensing images are used as the visual input, and phenotypic text descriptions of pine wilt disease (PWD) are further added to form cross-platform multimodal inputs; thus, the model’s capacity to represent high-level semantic features of the disease is enhanced, as shown in Figure 3. Subsequently, a Cross-Platform Semantic Consistency Enhancement (SCE) module is added at the high-level feature extraction stage, as shown in Figure 4. This module improves the consistency of high-level semantic representations for diseased targets in UAV and satellite images and, by means of a collaborative reinforcement mechanism for key discriminative features, further enhances the model’s capacity to learn discriminative information of cross-platform disease targets. In addition, after high-level semantic enhancement, a Spurious-Correlation Suppression Relational Modeling module (SSRM) is introduced, as shown in Figure 5, to explicitly model the relational dependencies among disease target regions and background contextual regions, and thus to suppress spurious correlation interference caused by complex backgrounds to improve the detection accuracy of real disease targets. Finally, a Progressive Phenotype Adaptation Mechanism (PPAM) is put forward, as shown in Figure 6. Therefore, a progressive task sequence has been built based on the evolutionary pattern of PWD phenotypes, from obvious to ambiguous, and a collaborative optimisation strategy for historical knowledge retention and new knowledge acquisition has been applied to further improve the continuous adaptability of the model to weak-phenotype samples and cross-stage complex samples. SCPA-Net has generally maintained the efficiency of the detection framework and, at the same time, exhibits strong capabilities in cross-platform semantic consistency modeling, suppression of complex background interference, and adaptation to weak-phenotype samples; therefore, it provides good support for the accurate detection of pine wilt disease tree crown targets.

#### 2.2.1. Cross-Platform Semantic Consistency Enhancement (SCE) Module

The second challenge lies in semantic inconsistency between UAV and satellite platforms in cross-platform PWD detection tasks. Given that the spatial resolution, observation scale, imaging perspective and background complexity of the two platforms are significantly different in the case of joint UAV and satellite detection, the same diseased tree crown target will generally exhibit different texture details, local structures and semantic response patterns across these two platforms. Therefore, the model will be unable to create a unified and stable high-level semantic representation. When the diseased area and the background area have similar colours, textures and shapes, and the weak-phenotype diseased trees and healthy crowns are also close to each other in feature space, the traditional detection model cannot distinguish between them well. As a result, problems such as semantic misalignment, response drift and degradation of cross-platform generalisation ability may occur, thus reducing detection accuracy.

In recent years, many researchers have put forward various ways to address the problem of feature distribution shifts under cross-domain and cross-modal conditions. Xu et al. put forward a Multi-Level Alignment Network to reduce the detection bias between optical remote sensing and SAR by using multiple levels of domain alignment strategies, and it has been shown that multi-level feature consistency modelling helps improve cross-domain remote sensing detection [21]. Chen et al. have also put forward a method for domain adaptation in remote sensing object detection based on adversarial learning. Aligning the feature distributions of the source and target domains helps to reduce domain shift in cross-scene situations effectively [22]. Zhao et al. proposed YOLO-CMFM for visible-SAR multimodal remote sensing object detection. In order to improve the integration of all kinds of data through edge guidance and gated cross-attention, a hybrid model consisting of explicit alignment and attention-based fusion has been proposed to enhance the robustness of representations in the presence of adverse conditions [23]. Although the above methods have achieved some results in cross-domain or cross-modal remote sensing tasks, they are still facing challenges in dealing with high-level semantic shifts of pine wilt disease targets under UAV and satellite conditions. The previous ways have been unable to build consistent and reliable semantic representations for the disease. Under weak phenotypic conditions and complex backgrounds, the aligned key discriminative regions and important feature channels still lack targeted collaborative enhancement mechanisms, and thus, the model’s performance in cross-platform detection is limited.

To alleviate the problem of high-level semantic shifts caused by significant differences between UAV and satellite remote sensing imagery in terms of spatial resolution, observation scale, imaging perspective, and background complexity, this paper proposes a Cross-Platform Semantic Consistency Enhancement (SCE) module. Different from methods that simply attach enhancement structures after high-level features, SCE adopts a progressive three-stage alignment strategy. It divides cross-platform high-level semantic consistency modeling into three consecutive stages: initial semantic latent space construction, cross-platform joint alignment, and detection-oriented refinement. Based on this alignment framework, this paper further incorporates high-level visual semantic adaptation, key discriminative feature enhancement, visual–text consistency constraints, and text-guided detection feature modulation. Through this design, the high-level semantic gap between different platforms is progressively reduced, and the model’s ability to respond to disease-related discriminative information is improved.

Let the input high-level visual feature to this module be denoted as $X\in R^{B\times C\times H\times W}$, where B denotes the batch size, C denotes the number of channels, and H and W represent the spatial dimensions of the feature map. To avoid introducing excessive disturbance to the detection backbone by directly imposing strong semantic constraints on the original high-level features, a lightweight semantic adaptation mapping is first applied to the input features. The computation is formulated as shown in Equation (1).

(1) $$X_{a}=\phi (BN(Conv_{1\times 1}(X)))$$

where ${Conv}_{1\times 1}(\cdot )$ denotes the channel mapping operation, BN(⋅) denotes batch normalization, and ϕ(⋅) denotes the SiLU activation function. This process does not reconstruct platform distributions through complex transformations. Instead, it reorganizes high-level visual responses using low-cost channel reconfiguration, enabling the features to obtain a representation more suitable for subsequent alignment and enhancement while maintaining the stability of the original topological structure. After obtaining the intermediate aligned feature $X_{a}$, this paper further employs a dual-attention mechanism to collaboratively enhance the key discriminative features of disease targets. First, channel attention is applied to model the importance of different semantic channels. Specifically, global average pooling and global max pooling are performed on $X_{a}$ to obtain two sets of channel-wise descriptor vectors. These descriptors are then passed through a shared bottleneck mapping to learn inter-channel dependencies, followed by a Sigmoid activation to obtain channel weights, as shown in Equation (2). Where σ(⋅) denotes the Sigmoid activation function, and $MLP\left(\cdot \right)$ consists of two 1×1 convolution layers with a ReLU activation in between. Based on the obtained channel weights, channel-wise reweighting is applied to the input features to produce channel-enhanced features, as formulated in Equation (3).

(2) $$M_{c}=\sigma (MLP(AvgPool(X_{a}))+MLP(MaxPool(X_{a})))$$

(3) $$X_{c}=X_{a}⊙M_{c}$$

where ⊙ denotes element-wise multiplication. This step highlights high-response semantic channels that are more relevant to pine wilt disease (PWD) tree crown discrimination, while suppressing ineffective responses caused by background noise, platform differences, and redundant textures. Subsequently, based on the channel-enhanced features, spatial attention is further introduced to emphasize the key regions where diseased targets are located. Specifically, average response maps and maximum response maps are computed along the channel dimension for $X_{c}$, and then concatenated along the channel dimension. The concatenated features are passed through a convolution mapping to generate spatial attention weights, as shown in Equation (4). Where $\left[\cdot ,\cdot \right]$ denotes channel concatenation, ${Avg}_{c}(\cdot )$ and ${Max}_{c}(\cdot )$ denote average aggregation and max aggregation along the channel dimension, respectively, and $f_{7\times 7}(\cdot )$ denotes a 7×7 convolution mapping function. Based on this, the spatially enhanced features can be obtained as formulated in Equation (5).

(4) $$M_{s}=\sigma (f_{7\times 7}([Avg_{c}(X_{c}),Max_{c}(X_{c})]))$$

(5) $$X_{s}=X_{c}⊙M_{s}$$

Channel attention mainly addresses the problem of “which semantic channels are more important,” while spatial attention focuses on “which spatial regions deserve more attention.” The two are clearly complementary. Therefore, they can further enhance the discriminative representation of disease targets on top of high-level semantic adaptation. To maintain the stability of the original high-level semantic structure while enhancing key features, a residual connection is finally adopted to construct the output feature, as shown in Equation (6).

(6) $$X_{e}=X+X_{s}$$

The core idea of this design is not to replace the original high-level semantics, but to preserve the main structural information while re-injecting the key disease-related responses extracted by the enhancement branch into the main feature stream in a residual manner, thereby achieving more robust feature enhancement. Relying solely on the visual branch for cross-platform adaptation still makes it difficult to explicitly constrain the high-level semantic consistency of disease targets across different platforms. Therefore, this paper further introduces disease phenotype text descriptions as auxiliary semantic references. Let the text description of the input sample be denoted as text. First, its semantic features are extracted through a text encoder, and then a linear projection layer is used to map the text features into a unified semantic space, resulting in the text semantic vector as shown in Equation (7).

(7) $$T=W_{t}f_{BERT}(text)$$

where $f_{BERT}(\cdot )$ denotes the text encoding function, and $W_{t}$ denotes the linear projection matrix. This process enables the text branch to provide a relatively stable auxiliary semantic reference, offering high-level phenotypic constraints for visual semantic alignment. Meanwhile, high-level visual semantic representations are extracted from the enhanced visual features. Specifically, global average pooling is applied to the enhanced feature $X_{e}$, and then a visual projection matrix is used to map it into a shared semantic space consistent with the text representation, resulting in the visual semantic vector as shown in Equation (8). Where $W_{v}$ denotes the visual projection matrix, and GAP(⋅) denotes the global average pooling operation. Subsequently, a cosine consistency loss is employed to constrain the distance between visual semantics and text semantics in the shared space, as shown in Equation (9).

(8) $$V=W_{v}\;GAP(X_{e})$$

(9) $$L_{align}=1-cos(Norm(V),Norm(T))$$

where Norm(⋅) denotes the vector normalization operation. This loss serves as an explicit high-level semantic consistency constraint, guiding diseased tree crowns from different platforms to maintain more stable and consistent representations in the shared semantic space, rather than directly replacing visual representations with textual semantics. Instead, textual semantics serve as auxiliary semantic guidance to enhance cross-platform semantic consistency and improve discriminative representation learning for weak-phenotype disease targets. In addition to serving as a high-level semantic consistency supervision signal, textual semantics further participate in detection feature modulation. Let the visual feature representation before entering the detection head be denoted as $F_{d}\in R^{B\times C_{d}\times H_{d}\times W_{d}}$. First, the text semantic vector T is fed into the text enhancement branch to obtain the text modulation representation $T_{m}$. Meanwhile, global average pooling is applied to the visual detection feature $F_{d}$ to extract the image-level contextual representation $G_{d}$. After completing alignment along the channel dimension, the two are added together and passed through a Sigmoid activation to generate channel modulation weights, as shown in Equation (10). Then, channel-wise recalibration is applied to the detection features in a residual manner, as shown in Equation (11).

(10) $$M_{t}=\sigma (T_{m}+G_{d})$$

(11) $$F_{d}^{\prime }=F_{d}+F_{d}⊙M_{t}$$

Different from approaches that use text only as an additional supervision signal, this design allows textual phenotypic semantics to directly participate in the update process of detection features. In this way, the model’s response to disease-related semantic channels is further enhanced, thereby improving its discriminative capability under weak phenotypic conditions and complex background scenarios. Therefore, from the perspective of the complete data flow, the SCE module consists of four components: lightweight high-level visual feature adaptation, channel–spatial dual-attention collaborative enhancement, visual–text semantic consistency constraint, and text-guided detection feature modulation. The joint optimization objective of these components can be formulated as shown in Equation (12).

(12) $$L=L_{det}+\lambda_{align}L_{align}$$

where $L_{det}$ denotes the basic detection loss, and $\lambda_{align}$ denotes the weight of the semantic alignment loss. It should be further emphasized that the core of SCE is not limited to structural-level semantic enhancement, but lies in its adoption of a progressive three-stage alignment strategy to achieve cross-platform high-level semantic consistency modeling. In the initial semantic latent space construction stage, by prioritizing the optimization of semantic adaptation and output-related components, a preliminary shared semantic representation of disease targets across platforms is established. In the cross-platform joint alignment stage, overall collaborative optimization is further performed, allowing high-level visual representations and semantic consistency constraints to jointly function, thereby continuously reducing semantic discrepancies between different platforms. In the detection-oriented refinement stage, based on the established cross-platform semantic consistency, further refinement is conducted at the output end to improve the quality of the final detection representation and the stability of discrimination. Through the combination of this structural design and progressive alignment strategy, SCE is able to systematically unify the high-level semantic representations of pine wilt disease targets under UAV and satellite conditions, and further strengthen key discriminative regions and important semantic channels. As a result, it provides more stable and more discriminative high-level feature representations for the subsequent detection head.

#### 2.2.2. Spurious-Correlation Suppression Relational Modeling Module (SSRM)

In cross-platform remote sensing detection tasks for pine wilt disease (PWD), spurious correlation responses induced by complex backgrounds are one of the key factors affecting detection accuracy and robustness. In particular, in forest scenes, diseased tree crown targets often co-occur with shadows, bare land, healthy tree crowns, fallen branches and leaves, as well as other regions with similar textures, resulting in strong co-occurrence relationships between target regions and background contexts. In such cases, the model tends to treat background patterns that co-occur with diseased targets but are not true discriminative cues as effective features, thereby forming spurious correlation representations. For obvious disease targets, this problem is usually manifested as local false detections or enhanced background responses. For weak-phenotype diseased trees, ambiguous samples, and cross-platform noisy samples, this background co-occurrence interference is further amplified, making the model more prone to target–background confusion, unstable responses, and degradation of generalization performance.

In recent years, researchers have begun to address spurious correlation issues and insufficient relational representation in complex remote sensing scenarios from the perspectives of graph-based modeling and causal learning. For example, Liu et al. proposed the Global Heterogeneous Graph Convolutional Network (GHGCN), which models long-range dependencies between coarse-grained and fine-grained objects by constructing heterogeneous graph relationships, demonstrating that graph-based modeling helps enhance structural relational representation in complex remote sensing scenarios [24]. Meanwhile, Zhao et al. proposed CICRL-FLM, which analyzes misclassification problems in fine-grained landslide mapping from the perspectives of correlation and causality. By introducing counterfactual and causal representation learning mechanisms, their method effectively reduces spurious correlation interference induced by contextual co-occurrence [25]. Furthermore, Wang et al. proposed a Dual Causal-Aware Detection Transformer, which introduces causal-aware mechanisms into the remote sensing object detection framework. By explicitly modeling causal relationships, the method enhances detection reliability in complex scenarios [26]. Although the above studies have made certain progress in relational modeling and causal suppression, most existing methods are designed for general remote sensing classification, change detection, or environmental monitoring tasks, and still lack dedicated modeling for the complex coupling relationship between “target regions” and “background contexts” in pine wilt disease tree crown detection. Therefore, in complex forest environments, pine wilt disease targets are still easily affected by shadows, bare land, and healthy tree crowns, leading to spurious correlation representations induced by background co-occurrence, which limits the model’s stable discriminative capability for diseased targets.

To address this issue, this paper proposes a Spurious-Correlation Suppression Relational Modeling module. Instead of simply treating background suppression as attention filtering or convolutional enhancement, this module is jointly designed around three objectives: “target–context relationship decoupling,” “spurious correlation intervention suppression,” and “progressive stable optimization.” Specifically, the input high-level visual features are first decomposed into a target semantic branch and a context semantic branch, enabling the model to explicitly distinguish the different roles of potential diseased regions and background regions within a unified feature space. Then, heterogeneous relational propagation is employed to model multi-type interaction relationships between target nodes and context nodes, so as to obtain more comprehensive structural representations. On this basis, an observation–intervention dual-branch causal inference mechanism is further introduced to reduce spurious correlation responses induced by background co-occurrence. Finally, the causally enhanced results are re-injected into the original high-level feature map as correction signals, thereby enhancing the model’s focus on true disease-related discriminative cues while maintaining the stability of the backbone visual structure.

Let the input high-level feature map be denoted as $X\in R^{B\times C\times H\times W}$, where B denotes the batch size, C denotes the number of channels, and H and W represent the spatial dimensions. First, the input features are mapped into a target feature map $X_{t}$ and a context feature map $X_{c}$ through two parallel projection branches, as shown in Equation (13). Where $f_{t}(\cdot )$ and $f_{c}(\cdot )$ denote the target projection function and the context projection function, respectively. This operation is not a simple duplication of the input features. Instead, it maps the same high-level visual representation into two subspaces with different semantic focuses: the former is more oriented toward potential diseased target regions, while the latter is more oriented toward background context regions. Through this explicit branching design, the model is able to distinguish the different functional roles of “target semantics” and “background semantics” in the subsequent relational propagation process. After obtaining the target feature map and the context feature map, to avoid introducing excessive redundant nodes by directly constructing a dense graph structure over the entire feature map, representative node selection is further performed on both feature maps. Specifically, a target score map and a context score map are constructed, and a set of the most representative nodes is selected from spatial locations based on response strength, forming the target node set $H_{t}$ and the context node set $H_{c}$, as shown in Equation (14).

(13) $$X_{t}=f_{t}(X),X_{c}=f_{c}(X)$$

(14) $$H_{t}=TopK(X_{t},S_{t}),H_{c}=TopK(X_{c},S_{c})$$

where $S_{t}$ and $S_{c}$ denote the target score map and the context score map, respectively. The purpose of this step is to transform continuous spatial features into more compact and more discriminative node representations, so that the subsequent relational propagation focuses on high-response disease regions and key background regions, rather than performing uniform modeling over all spatial locations, thereby improving the efficiency of relational reasoning and the quality of representation. In the heterogeneous relational modeling stage, SSRM treats target nodes and context nodes as two types of nodes with different semantic meanings, and explicitly models four types of relational propagation: target-to-target, context-to-target, target-to-context, and context-to-context. Unlike conventional homogeneous graph propagation, this approach does not assume that all nodes interact in the same manner, but instead learns differentiated propagation mappings for different types of relationships. Therefore, the update of target nodes depends not only on the internal consistency relationships among target nodes, but also on the influence of background context on the target nodes. Similarly, the update of context nodes considers both their own internal structure and the reverse effect of target regions on context representations. This process can be expressed in a unified form, as shown in Equation (15).

(15) $${\tilde{H}}_{t}=\Psi_{t}(H_{t},H_{c}),{\tilde{H}}_{c}=\Psi_{c}(H_{c},H_{t})$$

where $\Psi_{t}(\cdot )$ and $\Psi_{c}(\cdot )$ denote relation-specific heterogeneous graph propagation functions. Through this design, the model is able to simultaneously model the semantic consistency within diseased targets, the contextual dependencies among background regions, and the interaction coupling between targets and backgrounds, thereby obtaining a more comprehensive structural relational representation. Furthermore, graph message passing is not completed in a single step, but gradually updates the representations of target nodes and context nodes through multi-step propagation, allowing the relational dependencies between them to be accumulated and dynamically refined layer by layer. However, relying solely on relational propagation is still insufficient to solve the spurious correlation problem, because some background regions, although highly correlated with diseased targets, are not the true causes that lead to the final detection results. Therefore, this paper further introduces interventional causal inference after heterogeneous relational modeling. First, global aggregation is performed on the updated target nodes and context nodes, respectively, to obtain the global target representation $g_{t}$ and the global context representation $g_{c}$, as shown in Equation (16).

(16) $$g_{t}=Mean({\tilde{H}}_{t}),g_{c}=Mean({\tilde{H}}_{c})$$

Subsequently, the global context representation is regarded as an important carrier of potential confounding factors, and the confounding representation z is estimated accordingly, as shown in Equation (17), where $f_{z}(\cdot )$ denotes the confounder estimation function. On this basis, an observation branch and an intervention branch are constructed. The observation branch is used to describe the response under natural observation conditions when the target and background jointly act, as shown in Equation (18), where $\left[\cdot ,\cdot \right]$ denotes the feature concatenation operation, and $f_{o}(\cdot )$ denotes the mapping function of the observation branch. Meanwhile, to characterize the target response after removing the influence of background confounding factors, the confounding representation is first projected, and then subtracted from the target representation to obtain the intervention-adjusted target representation, as shown in Equation (19). The intervention response is then obtained through the intervention branch, as shown in Equation (20).

(17) $$z=f_{z}(g_{c})$$

(18) $$y_{obs}=f_{o}([g_{t},g_{c}])$$

(19) $$g_{t}^{do}=g_{t}-f_{p}(z)$$

(20) $$y_{do}=f_{d}(g_{t}^{do})$$

where $f_{p}(\cdot )$ denotes the confounder projection function, and $f_{d}(\cdot )$ denotes the mapping function of the intervention branch. The key idea here is not simply to subtract the background, but to construct dual responses under observational and interventional conditions, enabling the model to distinguish which discriminative information originates from the diseased target itself and which is a spurious response induced by background co-occurrence. After obtaining the observation response $y_{obs}$ and the intervention response $y_{do}$, the difference between the two is further regarded as an approximation of the causal effect, as shown in Equation (21). Then, the intervention response and the causal effect are jointly fused to obtain the final causally enhanced representation, as shown in Equation (22).

(21) $$e=y_{obs}-y_{do}$$

(22) $$h_{c}=f_{f}([y_{do},e])$$

where $f_{f}(\cdot )$ denotes the causal effect fusion function. The significance of this design lies in the following: using only the observation response retains too much background-related information, while using only the intervention response may lose part of the contextual auxiliary information. By jointly modeling the two, the module is able to preserve both the stable discriminative components of the target and the bias information caused by background confounding, thereby forming a more robust high-level semantic representation. Finally, in order to apply the above causal inference results back to the visual feature map, the causally enhanced representation is not directly used to replace the original features. Instead, it is re-injected into the input features as a causal correction signal. Specifically, the causally enhanced representation is first mapped into a channel-wise bias term, and then combined with the input feature map to generate spatial gating. The original feature map is finally updated in a residual manner, as shown in Equation (23).

(23) $$X^{\prime }=X+\alpha \cdot G(X)⊙f_{h}(h_{c})$$

where $f_{h}(\cdot )$ denotes the channel projection function, G(⋅) denotes the spatial gating function, and α is a learnable scaling coefficient. The advantage of this design lies in that the module does not disrupt the original visual backbone structure. Instead, through a combination of “spatial selection + channel correction,” the causally enhanced information is flexibly injected into the original feature map, enabling high-level features to better respond to true disease-related discriminative information while preserving the original visual structure.

It should be further emphasized that SSRM is not composed of a static relational reasoning module alone, but gradually achieves spurious correlation suppression and structural relational optimization through a three-stage training strategy. In the first stage, the model primarily establishes basic relational representations between target nodes and context nodes, allowing the network to first acquire a relatively stable understanding of target–background structural relationships. In the second stage, heterogeneous relational propagation and spurious correlation suppression are further strengthened, enabling the model to gradually learn to distinguish true disease-related discriminative cues from background co-occurrence noise under complex background and cross-platform perturbation conditions. In the third stage, based on the already established stable relational structure, the high-level causal correction results and detection discriminative representations are further jointly refined, thereby improving the stability and robustness of the final detection outputs. SSRM progressively optimizes the model through three stages: basic relational modeling, spurious correlation suppression, and detection-oriented refinement. This progressive training strategy enhances the model’s ability to represent complex coupling relationships between disease targets and background contexts. In addition, SSRM explicitly reduces spurious correlation interference under complex forest environments, weak-phenotype conditions, and cross-platform noise. As a result, the module provides more stable and discriminative high-level semantic representations for the subsequent detection head.

#### 2.2.3. Progressive Phenotype Adaptation Mechanism (PPAM)

In cross-platform remote sensing detection tasks for pine wilt disease (PWD), disease phenotypes are not static, but exhibit a progressive evolution pattern from obvious to ambiguous as the disease progresses. For trees in the middle and late stages, the target regions often show strong color degradation, structural abnormalities, and clear boundaries, making them easier for detection models to learn. In contrast, for early-stage weak-phenotype diseased trees, the affected regions usually exhibit slight yellowing, local chlorosis, blurred boundaries, and texture responses highly similar to healthy crowns, which significantly increases the detection difficulty. If samples from different disease stages are simply mixed and trained in a single static process, the model tends to prioritize fitting the more obvious samples, while insufficiently learning early-stage weak-phenotype samples. On the other hand, when the model is further optimized for weak-phenotype samples, it may easily disrupt the previously learned discriminative knowledge for middle- and late-stage diseases, leading to a clear phenomenon of catastrophic forgetting.

In recent years, continual learning has been gradually applied to alleviate catastrophic forgetting in visual tasks. In remote sensing continual learning scenarios, Yu et al. proposed a dual-knowledge distillation incremental scene classification method. By jointly applying feature map distillation and response distillation to constrain the training process of new stages, their method reduces the model’s overfitting to new tasks while preserving existing knowledge, demonstrating that distillation mechanisms play a positive role in cross-stage knowledge retention [27]. Meanwhile, Sun et al. proposed a Dual Domain Control via Active Learning framework for incremental object detection in the remote sensing domain. By selecting and retaining representative historical samples through active learning and combining cross-domain feature alignment strategies, their method alleviates catastrophic forgetting during continual learning, indicating that sample-retention-based replay strategies remain valuable in cross-domain remote sensing detection tasks [28]. In addition, Zhang et al. proposed SCF-CIL, a multi-stage regularization method for SAR target class-incremental learning. By introducing parameter constraints and stability control mechanisms during the continual learning process, their method reduces the disruption of new tasks to the representation space of previously learned knowledge, demonstrating that regularization strategies can also be effective in remote sensing continual learning tasks [29]. Although the above studies have explored anti-forgetting strategies from the perspectives of distillation, sample retention, and parameter constraints, most existing methods are designed for general tasks such as scene classification, SAR target recognition, or domain-incremental detection. They still lack dedicated designs for the progressive evolution process of disease phenotypes from obvious to ambiguous in pine wilt disease detection scenarios. Therefore, in early disease stages and under weak phenotypic conditions, the model still finds it difficult to maintain stable memory of previously learned disease knowledge while adapting to new-stage samples, leading to knowledge forgetting and fluctuations in detection performance.

To address this issue, this paper proposes a Progressive Phenotype Adaptation Mechanism (PPAM). This mechanism does not treat the adaptation process as simply “training for more epochs,” but instead provides a unified design around four components: progressive task construction, historical experience replay, cross-task feature distillation, and parameter importance constraint. In this way, the model is able to learn progressively along the difficulty path from obvious to ambiguous PWD phenotypes, while retaining previously learned disease discriminative knowledge as much as possible during adaptation to subsequent weak-phenotype samples.

Specifically, PPAM does not construct tasks based on temporal order or random partitioning, but instead builds a progressive task sequence according to sample detection difficulty. For any input sample, this paper comprehensively considers its detection confidence, the matching quality between predicted bounding boxes and ground-truth boxes, the degree of missed detection, the proportion of small targets, and the phenotypic weakening information reflected in the text descriptions to construct a sample difficulty score, as shown in Equation (24). The coefficients 0.35, 0.20, and 0.10 were determined through preliminary hyperparameter tuning experiments on the validation dataset. Multiple candidate settings were evaluated, and the selected values provided a better balance between optimization stability and overall detection performance.

(24) $$d=0.35(1-\overset{\text{-}}{c})+0.35(1-\overset{\text{-}}{IoU})+0.20r_{miss}+0.10r_{small}+d_{text}$$

where $\overset{\text{-}}{c}$ denotes the average detection confidence, $\overset{\text{-}}{IoU}$ denotes the average matching IoU, $r_{miss}$ denotes the missed detection rate, $r_{small}$ denotes the proportion of small targets, and $d_{text}$ denotes the weak-phenotype difficulty term reflected by the text description. Subsequently, all samples are sorted in ascending order according to their difficulty scores and are divided into three task subsets, corresponding to late-stage obvious diseased trees, middle-stage transitional phenotype diseased trees, and early-stage weak-phenotype diseased trees. The purpose of constructing the task sequence in this way is to align the adaptation process with the evolutionary pattern of disease phenotypes from strong to weak, thereby avoiding learning disorder caused by random task partitioning. After the task sequence is constructed, PPAM does not simply perform sequential fine-tuning across tasks. Instead, it establishes a balance between new knowledge learning and old knowledge retention through joint optimization. For the current task batch $B_{t}$, the model first computes the basic detection loss on the current task, as shown in Equation (25).

(25) $$L_{det}=L_{det}(B_{t})$$

This loss corresponds to the standard detection objective on samples of the current stage and serves as the direct source of new knowledge learning. To avoid completely forgetting knowledge from previous tasks when learning subsequent tasks, an experience replay mechanism is introduced. Specifically, the system maintains a memory bank of historical samples. During the training of the current task, a number of historical samples are randomly sampled from this memory and jointly optimized together with the current task samples. If the replay sample set is denoted as $B_{r}$, then the corresponding replay loss can be written as shown in Equation (26).

(26) $$L_{rep}=L_{det}(B_{r})$$

The role of experience replay is to allow the model to periodically revisit earlier obvious samples and transitional samples while learning more difficult weak-phenotype tasks, thereby reducing the risk that old knowledge is completely overwritten by new samples. Different from simply extending the number of training epochs, experience replay explicitly reintroduces historical task information into the current optimization process, enabling the model to retain a certain level of memory of previous task distributions during the adaptation stage. However, experience replay alone is still insufficient to guarantee the stability of knowledge at the intermediate feature level. Therefore, this paper further introduces cross-task feature distillation. Specifically, before the training of each new task begins, the model from the previous stage is retained as a teacher model. During subsequent training, the current model is constrained such that its representations at several high-level feature layers do not deviate excessively from those of the teacher model. If the features of the teacher model and the current model at the selected layers are denoted as $\left\{F_{k}^{T}\right\}$ and $\left\{F_{k}^{S}\right\}$, respectively, then the distillation loss can be written as shown in Equation (27).

(27) $$L_{dist}=\frac{1}{K}\sum_{k=1}^{K}∥F_{k}^{S}-F_{k}^{T}{∥}_{2}^{2}$$

where K denotes the number of layers involved in distillation. Different from distilling only the final output, this intermediate feature distillation can more stably maintain the consistency of high-level semantic structures, thereby avoiding excessive feature drift when the model adapts to weak-phenotype tasks. Since the first two contributions have already established cross-platform semantic consistency and spurious correlation suppression capabilities in high-level features, the high-level distillation constraint enables PPAM to preserve these well-learned effective representations as much as possible when learning new-stage samples. To further prevent key parameters from being significantly altered during new task learning, this paper also introduces an Elastic Weight Consolidation constraint. After each task is completed, the importance of parameters is estimated based on the current task data, and a penalty is imposed during subsequent task training on the deviation of important parameters from their previously optimal values. The corresponding regularization term can be expressed as shown in Equation (28).

(28) $$L_{ewc}=\sum_{i}F_{i}(\theta_{i}-\theta_{i}^{*}{)}^{2}$$

where $F_{i}$ denotes the importance estimation of parameter $\theta_{i}$, and $\theta_{i}^{*}$ denotes the optimal parameter saved from the previous stage. Different from experience replay and feature distillation, EWC does not directly rely on historical samples or intermediate features, but suppresses excessive drift of key weights in subsequent tasks at the parameter level. Therefore, the three mechanisms are complementary in their working principles: experience replay is responsible for retaining historical sample distributions, feature distillation is responsible for maintaining high-level representation structures, and parameter constraints are responsible for stabilizing key parameters. By integrating these three mechanisms, the overall optimization objective of PPAM at each task stage can be expressed as shown in Equation (29).

(29) $$L=L_{det}+\lambda_{rep}L_{rep}+\lambda_{dist}L_{dist}+\lambda_{ewc}L_{ewc}$$

where $\lambda_{rep}$, $\lambda_{dist}$, and $\lambda_{ewc}$ denote the weight coefficients of experience replay, feature distillation, and parameter constraint, respectively. Through this joint optimization strategy, the model not only focuses on the detection performance of new-stage samples when learning the current task, but also explicitly considers the retention of knowledge from previous tasks.

In terms of training scheduling, PPAM does not activate all adaptation strategies simultaneously at once, but progressively introduces them according to task difficulty. For the easiest late-stage, obviously diseased tree task, the model first establishes basic disease discriminative capability. Then, in the middle-stage transitional phenotype task, experience replay, feature distillation, and parameter constraints are introduced to retain knowledge from the previous stage while absorbing new knowledge. Finally, in the early-stage weak-phenotype task, the above three mechanisms are continuously applied, and the participation of historical experience is further strengthened, so as to minimize forgetting of knowledge from the first two stages while adapting to the most difficult task. In this way, the adaptation process is no longer a simple sequential fine-tuning procedure, but is progressively carried out along the path of “late-stage obvious phenotype → middle-stage transitional phenotype → early-stage weak phenotype,” making the learning order consistent with the evolution pattern of disease phenotypes. In addition, PPAM does not operate independently from the first two contributions, but is built upon the foundation of cross-platform high-level semantic alignment and spurious correlation suppression that has already been established. In other words, the goal of the third contribution is not to train a completely new detector, but to further achieve continuous adaptation along the direction of increasing phenotypic difficulty while retaining the cross-platform semantic consistency and stable discriminative capability formed by the first two contributions. The advantage of this design is that the learning of subsequent weak-phenotype tasks does not need to start from scratch, but can be built upon the high-level semantic structures already established in previous stages, thereby improving the stability of the overall progressive adaptation process. Therefore, from an overall perspective, PPAM is not a simple combination of a single replay strategy or a single distillation strategy, but a continuous adaptation mechanism composed of progressive task construction, experience replay, cross-task feature distillation, and parameter importance constraints. Its key role is not to merely extend the training process, but to enable the model to learn progressively along the difficulty path of pine wilt disease phenotypes from obvious to ambiguous, while preserving as much as possible the disease-related discriminative knowledge established in previous tasks when introducing new weak-phenotype samples, thereby enhancing the model’s continual adaptation capability for weak-phenotype samples and cross-stage complex scenarios.

## 3. Experiments

### 3.1. Experimental Setting

All of the experiments in this study were carried out on the same hardware and software platform to ensure the accuracy of the results and prevent external interference. The hardware configuration of the experiment was provided by the AutoDL platform, and all experiments were conducted in the same computational environment to exclude the impact of differences in hardware. The same environment was used to develop the software, and the same version of the operating system and related tools was kept to prevent different versions of the software from causing result discrepancies. Table 1 shows the actual specifications of the hardware and software environment setup.

### 3.2. Evaluation Metrics Overview

Four representative indices of quality were selected to measure the performance of the proposed model in the experiment for pine wilt disease (PWD) target detection: Precision, Recall, F1-score, and mAP50. As shown in Equations (30)–(34), the above indicators are used to reflect the detection performance and localization accuracy of PWD objects in the model from several angles. TP is the number of PWD targets correctly identified by the model; FP is the number of areas that the model predicts as PWD targets but are not diseased; and FN is the number of PWD targets that are not detected by the model.

Precision: The ratio of the number of correctly predicted PWD targets to the total number of samples predicted as diseased targets; an index of detection accuracy. The formula for the above is:

(30) $$Precision=\frac{TP}{TP+FP}$$

Recall: The proportion of the actual PWD targets that have been correctly identified by the model, indicating the model’s coverage capability for disease targets. The formula of calculation is as follows:

(31) $$\;Recall=\frac{TP}{TP+FN}$$

F1-score: The harmonic mean of Precision and Recall, which can be used to evaluate the trade-off between detection accuracy and completeness of the target detection. The formula for the above calculation is as follows:

(32) $$\;F1=\frac{2\times Precision\times Recall}{Precision+Recall}=\frac{2TP}{2TP+FP+FN}$$

mAP_50_: Average Precision (AP) represents the average detection accuracy of a single category under an IoU threshold of 0.5, while mean Average Precision (mAP) represents the average of AP across all categories, which is an important metric for evaluating the overall performance of object detection models. Here, S denotes the total number of categories. The formulas are given as:

(33) $$\;AP=\int_{0}^{1}P(r)\;dr$$

(34) $$\;mAP=\frac{\sum_{j=1}^{S}AP(j)}{S}$$

Given that the main subject of this study is the detection of pine wilt disease, the above evaluation indicators will all be used to evaluate how well the model can achieve detection accuracy and target completeness in general; therefore, they can provide a reasonable basis for further comparative experiments and ablation studies.

### 3.3. Module Effectiveness Experiments

Table 2 Experimental comparison of the effectiveness of the SCE, SSRM, and PPAM modules. Group (a), Group (b), and Group (c) correspond to the comparative experiments for SCE, SSRM, and PPAM, respectively. The proposed methods are highlighted in bold.

#### 3.3.1. Effectiveness of the SCE

To solve the problem of high-level semantic inconsistency caused by differences in spatial resolution, observation scale and imaging perspective between UAV and satellite remote sensing images, this paper introduces a Cross-Platform Semantic Consistency Enhancement (SCE) module. The proposed module combines cross-platform high-level semantic consistency modelling with a key discriminative feature enhancement mechanism to improve the consistency and discriminability of semantic representations for pine wilt disease (PWD) targets across different platforms, and thus reduce semantic drift in cross-platform detection more effectively.

Cross-platform high-level semantic consistency is required for PWD detection, as the same diseased tree crown often has different texture details, local structures and contextual response patterns in UAV and satellite images. High-level semantic consistency modelling restricts the feature distributions of diseased targets from different platforms within a shared semantic space, and thus the model can learn more consistent disease representations by not relying solely on implicit feature adaptation in a single platform. For cases with weak phenotypes and complex backgrounds, an explicit semantic constraint can be added to reduce high-level semantic shifts due to platform differences and improve the stability of cross-platform disease target representations.

A high-level semantic-consistent model is built to further enhance the refined features in cooperation with a discriminative feature enhancement mechanism. Channel-wise enhancement is employed to adaptively increase the prominence of semantic channels that are more relevant to disease discrimination, and spatial-wise enhancement further amplifies key regions where diseased targets are located. In addition to the above semantic-consistency constraint, this collaborative enhancement mechanism strengthens and refines the main discriminative information at the feature level to help the model learn representations of subtle differences in diseased areas and complex background environments, while also maintaining strong high-level semantic coherence. Therefore, SCE reduces platform differences at the semantic level and enhances the discriminative ability of the model for disease targets at the feature level. In addition to the proposed SCE module, five other representative alignment modules from the past two years have also been included in the comparison: WMLFA [30], CPA [31], LFA [32], RlITA [33], and SAM [34].

As shown in Table 2, the quantitative results are as follows: the proposed SCE module achieves the best performance among all evaluation indicators, with a Precision of 65.20%, Recall of 63.61%, F1-score of 64.39%, and mAP50 of 62.65%. SAM, the best-performing method, has achieved the following values: 64.55%, 62.86%, 63.69% and 62.04%; RlITA is 64.39%, 62.61%, 63.49% and 61.87%, respectively. Based on the above experiments, the proposed SCE module performs better than other alignment modules in addressing the problem of cross-platform high-level semantic inconsistency. The excellent performance of SCE is mainly due to the combination of explicit high-level semantic consistency modeling and key discriminative feature enhancement; that is, the former helps the model learn stable cross-platform disease semantic representations, and the latter further strengthens the feature representation of key discriminative channels and salient regions. Therefore, the model will have good stability and reliability in a harsh environment for complicated cross-platform PWD detection.

#### 3.3.2. Effectiveness of the SSRM

To solve the problems of complex background interference and target-background spurious correlation responses in cross-platform remote sensing detection of pine wilt disease (PWD), this paper introduces a Spurious-Correlation Suppression Relational Modeling module (SSRM). Target-background relational modeling and a background confounding suppression mechanism have been combined in the proposed module to explicitly represent the complex dependency relationships among disease target regions and background contextual regions, and to further reduce the spurious correlation representations induced by background confounding factors; thus, the discriminative ability of the model for real disease targets has been enhanced.

Relational models are suitable for disease detection in tree crowns because areas with diseased tree crowns are often close to shadows, other healthy tree crowns, and other regions with similar textures. Construct a network of the target area and its surrounding areas (context), and then use SSRM to create a high-level relational graph that shows the connections among them more explicitly. Relational modelling has been used in the above module to adjust for background confounding factors, thereby separating true disease responses from those caused by spurious correlations in the presence of co-occurring backgrounds. Thus, the module can use structural relational information to reduce the negative impact of a complex background on the detection result further. In addition to the proposed SSRM module, five representative modules from the past two years have also been used for comparison: HSGI [35], HGC [24], AM-CCR [36], CAM [37], and CIGN [38].

As shown in the quantitative results in Table 3, the proposed SSRM has achieved the best performance among all evaluation indicators, with Precision, Recall, F1-score and mAP50 reaching 65.89%, 62.76%, 64.29 and 63.11%, respectively. CIGN is the best-performing method, with the following values: 64.26%, 62.58%, 63.41% and 62.07%, and CAM has values of 64.02%, 62.46%, 63.23% and 61.84%. The above results show that although the existing relational modelling module and the causal representation module can reduce false alarms in complex environments to a certain extent, neither of them, when used alone, can address the problem of interference from the background in PWD detection comprehensively.

As shown in Table 2, the improved performance of SSRM is mainly due to the combined effect of the two components: enhanced structural relational representation of target areas and background contexts through target-background relational modelling, and reduced interference from background confounding factors by a spurious correlation suppression mechanism. Therefore, SSRM shows better robustness and discriminative power for complex forest scenes and cross-platform PWD detection.

#### 3.3.3. Effectiveness of the PPAM

To solve the problems of knowledge decay and performance drop caused by the gradual change in disease phenotype from obvious to ambiguous in cross-platform remote sensing detection of pine wilt disease (PWD), this paper introduces a Progressive Phenotype Adaptation Mechanism (PPAM). The proposed mechanism includes progressive task construction, experience replay, cross-task feature distillation and parameter importance constraints to enhance the model’s continuous adaptation ability for disease samples with different phenotypic stages in continuous learning, mitigate the under-learning of weak-phenotype samples and the disruption of prior knowledge caused by subsequent task training.

Progressive task construction is required for PWD detection because disease phenotypes are not static; they change gradually from late-stage, obviously diseased trees to middle-stage transitional phenotypes and, finally, to early-stage weak phenotypes as the disease progresses. Divide the samples into three levels of difficulty, and subsequently have the model first learn relatively simple and obvious disease patterns, and then gradually learn more difficult weak-phenotype patterns; thus, the continuous learning process will align with the evolutionary laws of disease phenotypes. Thus, it is less likely that random task partitioning will cause a learning disruption and thus will support the foundation for the following continual learning strategies more reliably.

Progressive task partitioning has been used by PPAM to preserve prior knowledge in conjunction with experience replay, cross-task feature distillation and parameter importance constraints. Experience replay can be used to periodically retrieve samples from previous tasks during the learning of new tasks; Feature distillation can maintain the consistency of high-level representations across different stages of task learning to avoid excessive feature drift when adapting to new samples; and parameter importance constraints can limit the extent of update for essential parameters in subsequent tasks. By means of the above joint mechanism, PPAM can acquire new weak-phenotype knowledge and, at the same time, retain the previously established disease discriminant ability as much as possible. Therefore, the goals of the training activities are to extend the training period and to achieve a new equilibrium between retaining old knowledge and obtaining new knowledge in a state of continuous learning. In addition to the proposed PPAM mechanism, five representative continual learning methods from the past two years have also been compared: IAD [39], PASS [40], DDC-AL [28], SDC [41], and EWC [42].

As shown in Table 2, the quantitative results indicate that the proposed PPAM has achieved the best performance across all evaluation indices; that is, Precision, Recall, F1-score and mAP50 all reached their maximum values of 64.73%, 64.50%, 64.61% and 62.97%, respectively. Compared with the others, EWC is the closest to 64.73%, 63.71%, 64.22%, and 62.54%, and SDC is 64.58%, 63.55%, 64.06%, and 62.37%. DDC-AL, PASS and TAL-TD also perform poorly in addition. Based on the above results, it can be seen that while the current continuous learning methods have reduced the extent of forgetting to some extent, relying solely on distillation, experience replay, or only general continuous learning frameworks is still inadequate to address the problems that arise from progressive phenotype evolution in PWD detection.

The experimental results show that the performance improvement of PPAM mainly benefits from the integration of progressive task construction, experience replay, feature distillation, and parameter importance constraints. Progressive task construction enables the model to learn progressively from obvious symptoms to subtle disease characteristics. Experience replay ensures the continuous participation of historical task samples during training. Feature distillation maintains the consistency of high-level representations, while parameter constraints further reduce the drift of key knowledge during subsequent learning stages. PPAM is, therefore, a relatively good compromise between weak-phenotype learning and cross-stage knowledge retention to improve the robustness and stability of the model in complex continual learning situations.

### 3.4. Ablation Experiments

To ensure that the overall performance improvement of the model is not due to a single module or random fluctuations, and to verify the contributions of multimodal input, SCE, SSRM, and PPAM to the detection results, systematic ablation experiments have been conducted in this paper, and the results are shown in Table 3. The Precision, Recall, F1-score and mAP50 of the baseline model are 63.23%, 61.38%, 62.29% and 61.57%, respectively. Only adding multimodal input improved the four metrics to 63.97%, 62.03%, 62.98%, and 61.85%, respectively. Although the performance improvement introduced by multimodal input alone is relatively moderate, the results demonstrate that disease-related textual semantic information can provide auxiliary semantic guidance for disease target representation learning and enhance cross-modal semantic consistency. Furthermore, the textual semantic information establishes an important semantic foundation for subsequent SCE, SSRM, and PPAM optimization, indicating that text features play a measurable and positive role in improving the overall detection performance rather than serving only as supplementary information.

Based on the above evidence, the addition of SCE has improved the precision, recall, F1-score and mAP50 of the model to 65.20%, 63.61%, 64.39% and 62.65%, respectively; therefore, improving cross-platform semantic consistency can effectively reduce semantic mismatches among different platforms and enhance the discriminative ability for disease targets. SSR can also be used to create the above indicators; that is, 65.89%, 62.76%, 64.29% and 63.11%, so relational modelling combined with spurious correlation suppression can reduce the influence of a complex background significantly. When only PPAM is used, the results are 64.73%, 64.50%, 64.61% and 62.97%, respectively, and the increase is largest for Recall; therefore, it can be concluded that the progressive phenotype adaptation mechanism enhances the model’s ability to handle weak-phenotype samples and reduces false negatives.

All dual-module combinations are better than single-module configurations. SCE+SSRM is 67.31%, 65.57%, 66.43%, 64.10%, SSRM+PPAM is 66.47%, 67.39%, 66.92%, 65.26%, and SCE+PPAM is 68.66%, 66.51%, 67.57%, 64.58%. Based on the above results, the three proposed components show strong complementarity in cross-platform semantic consistency modelling, complex background interference suppression, and weak-phenotype adaptation.

When the three are used together for multi-modal input, SCE, SSRM and PPAM, the model performs the best; Precision, Recall, F1-score and mAP50 are all at 72.64%, 74.53%, 73.57% and 67.78%, respectively. Compared with the baseline, the above are improvements of 9.41%, 13.15%, 11.28% and 6.21%, respectively. The above results indicate that the three modules work together to achieve complementary enhancements in cross-platform semantic consistency, suppression of background spurious correlation, and weak-phenotype continual adaptation, thus improving the overall detection performance of the model significantly.

### 3.5. Comparison Experiments with Other Networks

Several other popular object detection models were also introduced in this experiment for comparison, including RT-DETRv3-R50 [43], D-FINE-L [44], DEIM-D-FINE-L [45], RTDETR-L [46], YOLOv12L [47], DEYO-L [48], and Mamba-YOLO-L [49]. The results of the experiment are shown in Table 4.

In the comparative experiments, the proposed model was also compared with the above advanced detection networks. As shown in Table 4, the proposed model has the best results for all the evaluation indicators, and Precision, Recall, F1-score, and mAP50 are all around 72.64%, 74.53%, 73.57%, and 67.78%, respectively. Some of the competing models have been superior in some individual indices; however, they are generally less effective than the proposed method.

RT-DETRv3-R50 has good global modelling in a real-time detection system. However, it is still lacking in fine-grained discrimination of disease targets in complex forest scenes, and the Precision, Recall, F1-score and mAP50 are as low as 63.37%, 64.78%, 64.07% and 56.57%, respectively. D-FINE-L has a strong fine-grained feature representation ability and achieved a relatively high precision and F1-score of 68.40% and 68.93%, respectively. However, its mAP50 is only 62.64%, and thus it is not suitable for all complex cross-platform situations. DEIM-D-FINE-L further improves feature modelling based on the above, but its mAP50 is only 58.53%, so it is not yet suitable for complex backgrounds and cross-platform variations. RTDETR-L has achieved a Recall of 65.26% and can cover the targets well; however, its Precision and mAP50 are only 63.82% and 63.84%, respectively, and thus do not meet the requirements set by the proposed method.

Among the popular YOLO series models, YOLOv12L is a relatively light-weight design with good detection performance, achieving a recall rate of 71.36%. However, its Precision and mAP50 are 64.51% and 62.12%, respectively, so it still has a large number of false detections in the presence of complex background interference and cross-platform variations in PWD detection. DEYO-L has a high Precision of 68.92%, showing good target discrimination ability, but its mAP50 is only 55.02%, and the overall detection performance is unstable. Mamba-YOLO-L has been used to build the model for long-range dependence, and it has achieved a recall rate of 73.77%, which is considered relatively high. However, its Precision and mAP50 are 66.37% and 62.57%, respectively, and its overall performance is still lower than that of the proposed method.

The above way of working is relatively better than the previous one. Therefore, the new network will not only use a single detection framework, but also simultaneously address the three problems mentioned above: improving cross-platform semantic consistency, handling complex background interference, and strengthening robustness for weak-phenotype samples. The SCE can be employed to reduce the difference in high-level semantics between UAV and satellite images and improve the consistency of disease target representations at both platforms. SSRM module strengthens the link between the target area and the background area, reduces damage caused by spurious correlation in complex backgrounds, and thus enhances the detection rate of real disease-related features for the model. The PPAM continuously optimises along the progressive path from obvious to ambiguous disease phenotypes, retains existing knowledge, and adapts to subsequent weak-phenotype samples to improve the generalisation capability of the model for complex data. Together, the three parts can achieve high accuracy, high recall, and stable overall performance in the proposed model for difficult cross-platform PWD detection problems.

**Figure 7:**
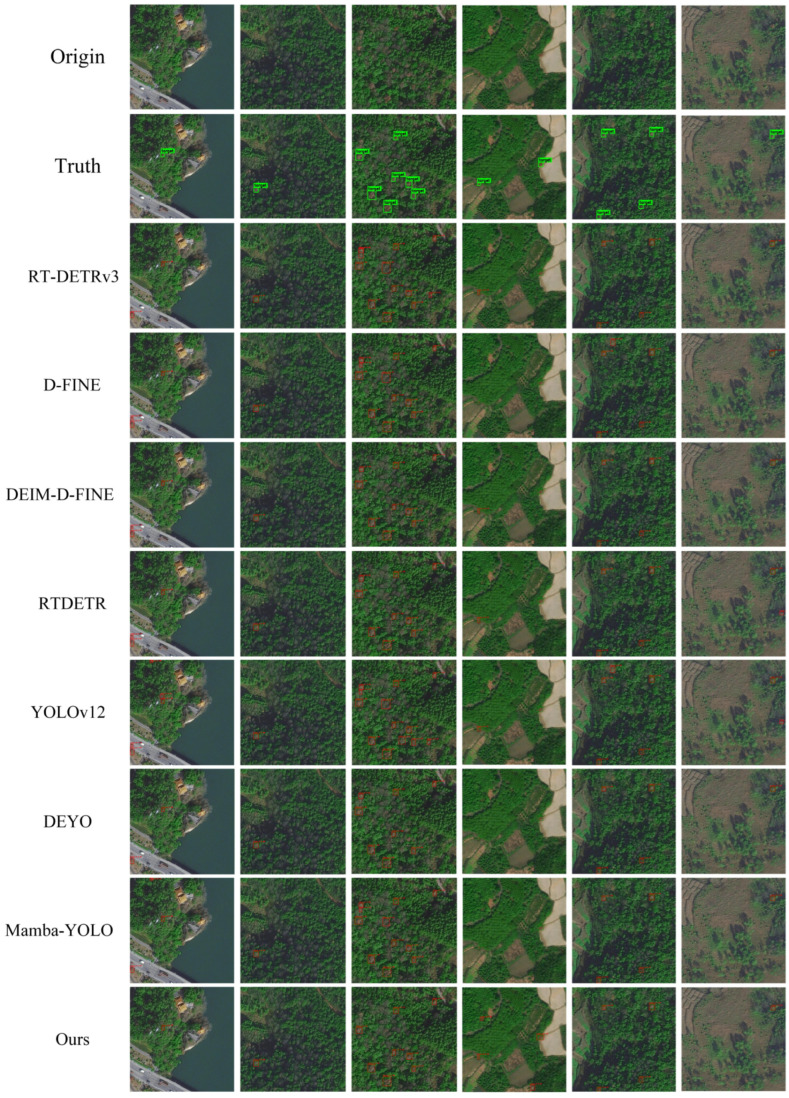
Comparison of detection results of different models in complex scenarios.

In short, based on the quantitative results in Table 4 and the visualization results in Figure 7, it can be seen that the proposed network has achieved the best overall performance among several other advanced detection models; thus, it shows superior robustness, discriminative ability, and generalization capability in cross-platform remote sensing detection of pine wilt disease.

### 3.6. Generalization Experiments

A generalisation test evaluates how well a model performs on unseen data after being trained and generalised from some training data. The above experiments show that the model has some strengths and weaknesses in different environments, such as different data quality, resolutions and light conditions, and its performance in complex background situations.

#### 3.6.1. Generalization Experiment of Satellite Dataset

To further verify the detection stability and cross-platform representation ability of the proposed method in a satellite scenario, cross-platform generalization experiments are conducted specifically for the satellite platform in this paper. It should be pointed out that this experiment is neither a pure satellite-only training and validation set nor a strict zero-shot cross-domain transfer. Therefore, a joint training set of UAV and satellite data was constructed, and the best-performing model weights were selected based on the satellite validation set. Finally, the model performance was evaluated using satellite data. An experimental system consistent with the objectives of this study was established, and its disease target recognition capability and generalization performance on the satellite platform under the cross-platform joint modeling framework were examined.

Satellite imagery has a relatively large observation scale compared to UAV images, is more prone to background interference, and has a weaker local texture response. Therefore, in the satellite case, the disease targets are more prone to a high-level semantic shift, attenuation of weak phenotype responses, and target-background confusion. Thus, the test in space can better reflect the general-purpose robustness and practicality of the model on different platforms. If the model can still maintain good detection performance under this condition, it suggests that the model does not rely too much on local texture patterns from a single platform and has learned more transferable and stable disease-discriminative representations.

**Figure 8:**
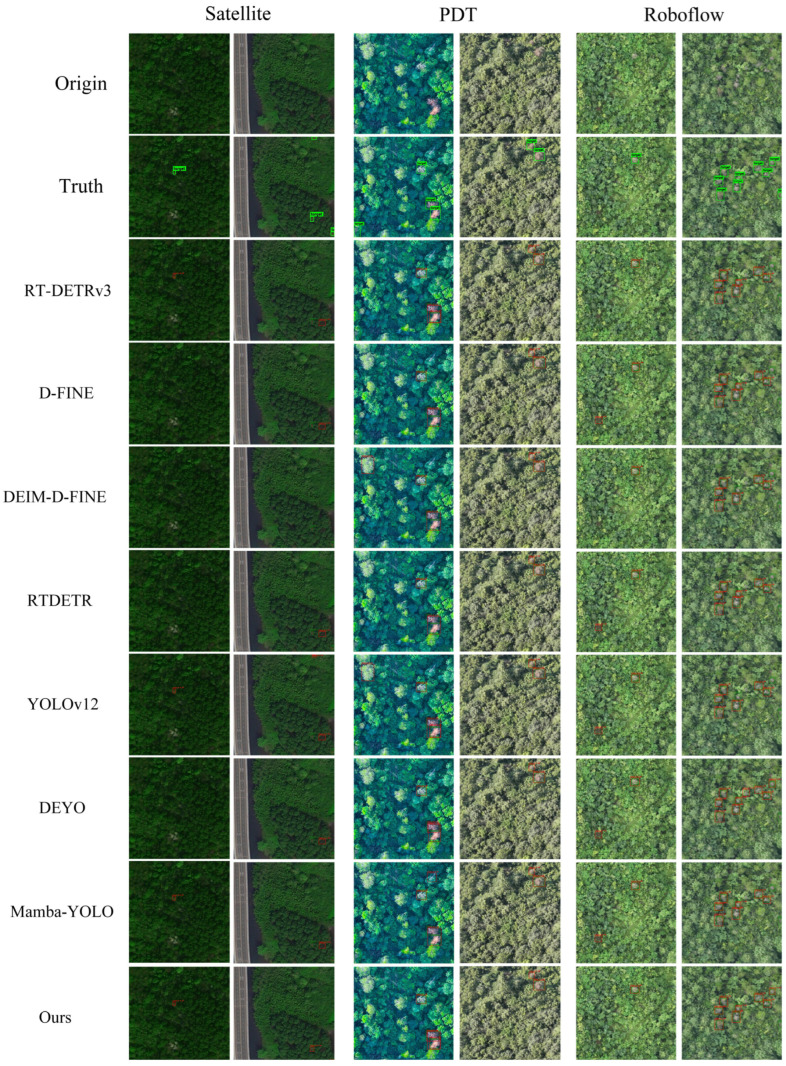
Comparison of detection generalization performance of different models in satellite and UAV imagery.

As shown in Table 5 and Figure 8, the proposed method has achieved the best detection results on the satellite dataset, with a Precision, Recall, F1-score and mAP50 of 73.32%, 62.38%, 67.41% and 53.93%, respectively. Mamba-YOLO-L is a better-performing competing method, and the proposed method increases Precision, Recall, F1-score, and mAP50 by 6.97%, 7.60%, 7.40%, and 5.27%, respectively. As shown in the above results, the proposed method has superior target discrimination and more stable detection performance in the satellite scenario.

As shown in the visualisation results in Figure 8, more precise location of the disease area and the reduction of false alarms caused by shadows, bare land and regions with similar textures have been achieved by the proposed method in challenging backgrounds, weak-phenotype targets and scenarios with large-scale variations. Some competing methods are more likely to have missed detections, false detections or unstable boundary localisation in satellite environments. Therefore, the above indicates that the new method is more accurate in terms of the quantitative index and also shows better stability and generality in real-world detection results.

The performance improvement of the proposed method on the satellite platform does not involve only a single indicator, but rather a general increase in precision, recall, F1-score, and mAP50. On the other hand, a relatively high Precision suggests that the model can suppress false detections in the presence of various sources of background noise more effectively. On the other hand, a higher Recall and F1-score indicate that weak-phenotype targets, blurred-boundary targets and targets with scale variations have been detected more effectively. At the same time, the considerable rise in mAP50 also shows that the proposed method can detect more actual diseased areas in satellite images and maintain a relatively high localization accuracy.

In short, the above results show that the collaborative design of the Cross-Platform Semantic Consistency Enhancement module, the Spurious-Correlation Suppression Relational Modeling module, and the Progressive Phenotype Adaptation Mechanism can improve the detection robustness and cross-platform generalization ability of the model for satellite scenarios.

#### 3.6.2. Generalization Experiment of PDT Public Dataset

To expand the generalisation range of the proposed method in a difficult background environment, experiments are also conducted on the PDT dataset. PDT is a high-precision UAV remote sensing dataset for detecting tree pests and diseases which aims to fill the deficiency of specialised datasets in forestry. UAVs have been used to acquire data from the air at about 200 metres high, and a variety of trees with pests and diseases have been recorded. The annotations are incorrect, and there are many complex scenes that are significantly different from real life. Overall, 400 images with pine wilt disease as targets were selected and annotated from the dataset in this study to assess the cross-dataset generalization ability of the model. The experimental results are as follows: Table 5.

As shown in Table 5, the proposed method has the best performance on the PDT dataset, with a Precision of 83.70%, a Recall of 79.73%, an F1-score of 81.67%, and an mAP50 of 75.68%. Compared with all other ways, the present one is relatively good in terms of detection accuracy, target recall and general performance. It can be seen that the model has good recognition performance in the original test cases and is also relatively stable and generalisable on other datasets.

RT-DETRv3-R50, D-FINE-L and RTDETR-L performed reasonably well on the PDT dataset; thus, it can be concluded that detection frameworks based on Transformer architectures or fine-grained distribution regression show some adaptability to high-altitude remote sensing applications. However, these methods are general visual feature models and do not have task-specific models for weak phenotypic features, local texture variations, and complex background interference in the target of pine wilt disease. Therefore, they have not improved significantly. DEIM-D-FINE-L and DEYO-L have achieved mAP50 values of 72.06% and 72.48%, respectively, and are relatively good in localization and matching. However, their performance is still lower than that of the proposed method; therefore, it can be concluded that only optimising the matching mechanism or dynamically modelling features is insufficient to address the problem of instability in disease target representation across different scenes.

YOLOv12-L, on the other hand, has achieved a relatively high Precision of 81.73% and is highly discriminative; however, its Recall is only 74.34%, indicating that some instances are still failing to be detected in complex backgrounds. Mamba-YOLO-L has a recall of 77.87% and an F1-score of 77.13%, performing relatively poorly overall; therefore, general detection frameworks based on state-space models are still not very suitable for fine-grained forestry disease detection in this context.

At the same time, this new way is also based on SCE, SSRM and PPAM to improve feature representation and detection performance by enhancing cross-platform semantic consistency, suppressing spurious correlations in complex backgrounds, and increasing adaptability to weak-phenotype samples.

#### 3.6.3. Generalization Experiment of Roboflow Pine Wilt Disease Dataset

More experiments will be conducted in the course of this work using the Roboflow dataset to assess how well it performs under circumstances of phenotypic ambiguity. This dataset was obtained from the Pine Wilt Disease Tree Computer Vision Dataset released on the Roboflow Universe platform by project-dkq3q. It is released under the CC BY 4.0 open-source license and is reasonably open and reproducible. The public page lists the datasets created for pine wilt disease tree target detection and may be used by others in their own research. Overall, 736 images and a single detection class were taken from the experimental version to test the generalisation capability of the model on a public dataset. The results of the experiment are as follows: Table 5.

As shown in Table 5, the proposed method also achieved the best results on the Roboflow dataset, with a Precision, Recall, F1-score, and mAP50 of 91.96%, 82.46%, 86.95%, and 80.55%, respectively. Among all the competing ways listed above, the proposed one has relatively good detection accuracy and a higher target recall rate. This shows that the model performs well with the self-constructed dataset and UAV scenarios, and is also relatively robust and generalizable under conditions of public datasets.

RT-DETRv3-R50, D-FINE-L, DEIM-D-FINE-L and RTDETR-L all achieve relatively high detection performance on this dataset; thus, it can be concluded that Transformer-based or fine-grained distribution regression-based detection frameworks are suitable for public forestry disease scenarios. Among them, DEIM-D-FINE-L has an mAP50 of 79.92% and shows good performance among the compared methods; D-FINE-L has an F1-score of 84.62% and is relatively well balanced in localization and classification. However, these methods are still relatively general in their learning of visual features and detection frameworks, and lack specific modelling capabilities for fine-grained phenotypic variations, cross-scene semantic changes, and difficult-to-obtain sample adaptations in pine wilt disease detection under complex background conditions. Therefore, their overall performance is not that of the above method.

RTDETR-L and YOLOv12-L have achieved higher precision values of 91.16% and 89.66%, respectively, and are thus more discriminating. However, their Recall values are only 77.14% and 78.45%, respectively; thus, there are some undetected instances in difficult environments and overall performance is constrained. Mamba-YOLO-L has achieved a precision, recall and F1-score of 88.61%, 80.81% and 84.53%, respectively, and is relatively balanced. However, its mAP50 is still lower than that of the proposed method; therefore, general detection frameworks based on state-space models have not yet been optimized for forestry disease scenarios. DEYO-L has a relatively lower all-around performance, with an F1-score and mAP50 of 78.91% and 71.51%, respectively; therefore, only dynamic feature modeling cannot fully address the complex representation of disease targets in public scenes.

At the same time, the new method has also improved upon the Roboflow dataset by adding SCE, SSRM and PPAM to enhance the capability of feature representation and detection robustness in three ways: strengthened cross-platform semantic consistency, reduced spurious correlations in complex backgrounds, and increased adaptability to weak-phenotype samples. SCE uniformly strengthens the high-level semantic representation of disease targets across multiple platforms and scenarios to reduce cross-scene semantic drift; SSRM improves the relational modelling of target regions and background regions, suppresses non-causal interference in complex backgrounds, and enhances discriminative stability in complicated environments; and PPAM gradually tunes the model during the evolution from obvious to ambiguous phenotypes to achieve robust generalisation of difficult samples and samples with weak phenotypes in public datasets.

## 4. Discussion

The problems of cross-platform Pine Wilt Disease target detection include changes in the scale of targets or increased background complexity, and more seriously, the fact that disease targets have both strong phenotype dependency and cross-platform semantic shifts. PWD generally does not show a stable and distinct structural contour in remote sensing applications; instead, it appears as progressive degradation phenotypes such as yellowing, browning and chlorosis. On the other hand, these phenotypes are easily changed by altering light, distance from the camera, canopy structure similarity, and interference from background vegetation. On the other hand, they have different high-level semantic distributions due to changes in spatial resolution and observation scale of UAV and satellite platforms. Therefore, this task cannot be solved efficiently by using only general visual features. Instead, it is a remote-sensing disease detection problem that jointly considers cross-platform high-level semantic inconsistency, spurious correlations caused by complex backgrounds, and the difficulty of continuous adaptation to weak-phenotype samples. This is also why many general-purpose detectors perform well on the standard benchmark but still have false positives and false negatives, and are less generalisable in this task.

Methodologically, the three modules of different sizes in SCPA-Net are designed to work together effectively to achieve this goal. First, SCE improves the consistency of high-level semantic representations for disease targets under UAV and satellite conditions, and further strengthens key discriminative channels and salient regions to reduce cross-platform semantic shifts. Second, SSRM cannot show the complicated relationships at the regional level and thus limits the impact of all kinds of external factors that have not been considered, leading to misleading results. PPAM is an adaptable task flow that considers the gradual changes in PWD phenotypes from obvious to ambiguous, maintains prior knowledge through historical experience retention and cross-stage feature stability, and sets key parameter constraints. It can reduce catastrophic forgetting during the learning of weak-phenotype samples. The three modules are to improve cross-platform semantic consistency, suppress spurious correlations in complex backgrounds, and continuously adapt to weak-phenotype samples; together, they have enhanced the overall performance of the model.

According to the experimental results, the three above ways of occurrence do not operate in isolation but can be used together. Single-module ablation experiments show that SCE, SSRM and PPAM all achieve stable performance improvement. Dual-module combinations also show strong complementarity among the modules. To further evaluate the effectiveness of SCPA-Net under cross-platform pine wilt disease detection scenarios, additional comparisons with representative pine wilt disease detection approaches were conducted, including SLMW-Net [50], TVIM [51], PWD-YOLO [52], YOLOv11-OC [53], and Pine-YOLO [54]. These methods mainly focus on UAV-based disease feature extraction, multimodal information utilization, or local representation enhancement. However, they generally lack dedicated designs for jointly addressing cross-platform semantic inconsistency, spurious correlation interference, and progressive adaptation to weak-phenotype disease stages.

As shown in Table 6, SCPA-Net achieved the best performance on the self-built cross-platform dataset, where UAV and satellite images were jointly used for training while satellite imagery was exclusively employed for validation. Specifically, SCPA-Net achieved 72.64% Precision, 74.53% Recall, 73.57% F1-score, and 67.78% mAP_50_, consistently outperforming representative pine wilt disease detection approaches. Compared with TVIM and SLMW-Net, which mainly focus on semantic enhancement and multimodal information utilization, SCPA-Net further improves semantic consistency under UAV–satellite joint scenarios. Compared with PWD-YOLO, YOLOv11-OC, and Pine-YOLO, which primarily strengthen local feature extraction and detection capability, SCPA-Net additionally considers spurious correlation suppression and continual adaptation to weak-phenotype disease samples. These results indicate that jointly modeling cross-platform semantic consistency, suppressing spurious correlations under complex forest backgrounds, and progressively adapting to disease phenotype evolution contribute to more robust disease representation learning and stronger cross-platform generalization capability.

Based on the excellent results in the main experiments, comparative tests and generalization experiments, it can be concluded that jointly considering cross-platform high-level semantic consistency, complex background spurious correlation suppression and progressive adaptation to weak-phenotype samples is required to enhance the detection accuracy of cross-platform PWD. In other words, the strength of the proposed method does not come from any single module but from the combined effect of the three innovations at the level of feature representation, relational modelling and adaptation strategy.

Although the new method has achieved some good results in experiments, it is not perfect either. First of all, to some extent, the superior performance of the model is still based on the presence of visible phenotypic characteristics of PWD in remote sensing images. Therefore, in situations where the early-stage disease symptoms are very mild, colour changes are slight, or easily mistaken for seasonal colour fluctuations in other trees, the discriminative ability of the current method will still be relatively weak. Second, although the text semantic branch, relational modeling with spurious correlation suppression, and progressive adaptation mechanism have improved detection accuracy and generalisation performance, they have also increased model complexity and inference cost, and may be unsuitable for direct deployment in resource-constrained devices and real-time forestry monitoring scenarios. Third, the current experimental data focus only on PWD-related situations. The coverage of other tree species, other forestry diseases and more complex imaging conditions is still limited, and thus the transferability of the model to other areas of forestry remote sensing needs to be further verified. Although SCPA-Net is currently developed and validated for pine wilt disease detection, its core mechanisms are not inherently restricted to a specific disease category. The proposed cross-platform semantic consistency enhancement, spurious correlation suppression, and progressive adaptation strategies mainly address challenges related to cross-platform semantic discrepancies, complex background interference, and weak target characteristics. Therefore, the proposed framework has the potential to be extended to other forestry disease monitoring tasks and remote sensing detection scenarios exhibiting similar characteristics. However, further validation on broader forestry datasets is still required in future work.

Future work will also be carried out in other places. First, lightweight network design, pruning, quantization and distillation-based model compression strategies can be used to reduce the model’s complexity and computational cost for deployment. Second, more auxiliary information can be added to the training data, such as multi-temporal remote sensing data, multispectral data and environmental variables (e.g., climate, soil moisture and terrain conditions), to improve the generalisation ability of the model. Third, to further increase the training data set in terms of type and scale, more tree species, more disease categories and more diverse imaging conditions should be added to enhance generalisation ability and cross-task transferability. Fourth, the pipeline for image preprocessing and data augmentation can be optimised under adverse circumstances such as poor lighting conditions, seasonal changes and low-quality remote sensing images, and thus increase the robustness of the model in real-world forestry monitoring. Fifth, to enhance the real-time performance and system integration capabilities of the model for practical applications in forestry inspection, disease early warning, and forest pest and disease control.

In short, the purpose of this paper is to develop a high-efficiency, stable and cross-platform generalizable remote sensing target detection system for pine wilt disease that can accurately identify disease targets and provide technical support for forestry inspection, early warning of diseases, and management of forest pests and diseases.

## 5. Conclusions

To solve the problems of cross-platform high-level semantic inconsistency, spurious correlations induced by complex backgrounds, and difficulty in continuous adaptation to weak-phenotype samples for cross-platform pine wilt disease (PWD) target detection, this paper introduces a Cross-Platform Semantic Consistency and Phenotype-Adaptive Detection Network named SCPA-Net. The two inputs for this method are remote sensing images and disease description text; a cross-platform multimodal detection model based on YOLOv10 is constructed. The three purposes of the above improvements are to enhance cross-platform semantic consistency, to reduce the interference of relations in complex backgrounds, and to gradually adjust to weak-phenotype samples. The SCE module can help to improve the consistency of high-level semantic representations for disease targets in UAV and satellite images, enhance the discriminative ability of key channels and salient regions, and thus reduce cross-platform semantic shifts. The SSRM module can directly model the relational dependency of disease target areas on background contextual areas in the SSRM module and suppress the effect of background confounding factors to reduce spuriously correlated responses in complex scenarios. PPAM builds an incremental task sequence based on the evolutionary pattern of disease phenotypes from obvious to ambiguous and regulates the learning process by jointly using historical experience retention, cross-stage feature stability, and key parameter constraints to improve the continuous adaptation ability of the model for weak-phenotype samples and cross-stage complex samples.

According to the above experimental results, the proposed method performs better in terms of module effectiveness tests, comparative studies and generalisation tests. Ablation studies have shown that all three proposed modules can improve the stability of performance, and thus they are effective for enhancing cross-platform semantic consistency, suppressing spurious correlations in complex backgrounds, and adapting to weak-phenotype samples. According to the above experiments, SCPA-Net has superior performance in several aspects over other popular object detection algorithms, including the precision, recall rate, F1 score and mAP@0.50. Generalization experiments also show that the proposed method has a high detection accuracy and stability in external datasets, and thus can be generalised to various conditions of forestry. Based on the above results, it can be seen that jointly improving the cross-platform high-level semantic consistency, complex background spurious correlation suppression, and progressive adaptation to weak-phenotype samples is an effective way to boost the performance of cross-platform PWD detection.

Overall, this work has improved the detection accuracy, cross-platform compatibility, and robustness in complex environments of the target identification of pine wilt disease in forestry remote sensing and offered a new methodology for cross-platform disease detection in forestry remote sensing. In the future, further optimisation will be carried out through the combination of lightweight model design, multi-temporal and multispectral information fusion, and the expansion of datasets to cover a larger area with more tree species and disease types to improve the deployment efficiency, as well as the adaptability and practical value, of real-world forestry monitoring systems.

## Figures and Tables

**Table 1:** Hardware and software parameters.

Hardware environment	CPU	AMD Ryzen 7 5800H
	GPU	NVIDIA GeForce RTX 3090
	RAM	64 GB
	Video Memory	24 GB
Software environment	OS	Windows 10 × 64
	CUDA Toolkit	V10.2
	CUDNN	V8.2.1
	Python	3.8

**Table 2:** Experimental comparison of SCE, SSRM and PPAM effectiveness.

Group	Model	Precision	Recall	F1-Score	mAP_50_
a	WMLFA	63.86%	62.05%	62.94%	61.28%
	CPA	63.34%	61.58%	62.45%	60.84%
	LFA	64.07%	62.26%	63.15%	61.53%
	RlITA	64.39%	62.61%	63.49%	61.87%
	SAM	64.55%	62.86%	63.69%	62.04%
	**SCE**	**65.20%**	**63.61%**	**64.39%**	**62.65%**
b	HSGI	63.21%	61.86%	62.53%	61.02%
	HGC	63.46%	62.17%	62.81%	61.29%
	AM-CCR	63.78%	62.34%	63.05%	61.57%
	CAM	64.02%	62.46%	63.23%	61.84%
	CIGN	64.26%	62.58%	63.41%	62.07%
	**SSRM**	**65.89%**	**62.76%**	**64.29%**	**63.11%**
c	TAL-TD	62.28%	62.74%	62.51%	61.66%
	PASS	63.26%	63.08%	63.17%	61.93%
	DDC-AL	62.16%	63.31%	62.73%	62.15%
	SDC	63.34%	63.55%	63.44%	62.37%
	EWC	63.68%	63.71%	63.70%	62.54%
	**PPAM**	**64.73%**	**64.50%**	**64.61%**	**62.97%**

**Table 3:** Results of ablation experiment.

Group	Multimodal Input	SCE	SSRM	PPAM	Precision	Recall	F1-Score	mAP_50_
①	-	-	-	-	63.23%	61.38%	62.29%	61.57%
②	√				63.97%	62.03%	62.98%	61.85%
③	√	√			65.20%	63.61%	64.39%	62.65%
④	√		√		65.89%	62.76%	64.29%	63.11%
⑤	√			√	64.73%	64.50%	64.61%	62.97%
⑥	√	√	√		67.31%	65.57%	66.43%	64.10%
⑦	√		√	√	66.47%	67.39%	66.92%	65.26%
⑧	√	√		√	68.66%	66.51%	67.57%	64.58%
⑨	√	√	√	√	72.64%	74.53%	73.57%	67.78%

√ indicates the presence of the corresponding module, while “-” indicates its absence.

**Table 4:** Comparative experiment results for different networks.

Network	Flops (G)	Parm (M)	Precision	Recall	F1-Score	mAP_50_
RT-DETRv3-R50	136	42	63.37%	64.78%	64.07%	56.57%
D-FINE-L	91	31	68.40%	69.47%	68.93%	62.64%
DEIM-D-FINE-L	95	34	67.37%	68.68%	68.01%	58.53%
RTDETR-L	125	47	63.82%	65.26%	64.53%	63.84%
YOLOv12L	88.9	26.4	64.51%	71.36%	67.76%	62.12%
DEYO-L	155	51	68.92%	65.95%	67.40%	55.02%
Mamba-YOLO-L	156.2	57.6	66.37%	73.77%	69.87%	62.57%
Ours	99.6	38.9	72.64%	74.53%	73.57%	67.78%

**Table 5:** Experimental results of generalization.

Group	Dataset	Model	Precision	Recall	F1-Score	mAP_50_
a	Satellite	RT-DETRv3-R50	64.78%	50.64%	56.84%	44.52%
		D-FINE-L	63.19%	49.62%	55.59%	42.65%
		DEIM-D-FINE-L	63.54%	48.25%	54.85%	43.32%
		RTDETR-L	64.83%	50.79%	56.96%	45.81%
		YOLOv12-L	66.25%	52.26%	58.43%	46.97%
		DEYO-L	65.42%	51.96%	57.92%	47.87%
		Mamba-YOLO-L	66.35%	54.78%	60.01%	48.66%
		Ours	73.32%	62.38%	67.41%	53.93%
b	PDT	RT-DETRv3-R50	78.14%	76.09%	77.10%	70.02%
		D-FINE-L	79.17%	75.36%	77.22%	70.38%
		DEIM-D-FINE-L	77.71%	77.25%	77.48%	72.06%
		RTDETR-L	78.38%	76.09%	77.22%	71.20%
		YOLOv12-L	81.73%	74.34%	77.86%	69.53%
		DEYO-L	78.76%	77.84%	78.30%	72.48%
		Mamba-YOLO-L	76.41%	77.87%	77.13%	70.61%
		Ours	83.70%	79.73%	81.67%	75.68%
c	Roboflow	RT-DETRv3-R50	81.48%	80.45%	80.96%	76.53%
		D-FINE-L	88.78%	80.85%	84.62%	78.18%
		DEIM-D-FINE-L	87.82%	81.84%	84.72%	79.92%
		RTDETR-L	91.16%	77.14%	83.57%	74.87%
		YOLOv12-L	89.66%	78.45%	83.68%	75.81%
		DEYO-L	85.11%	73.55%	78.91%	71.51%
		Mamba-YOLO-L	88.61%	80.81%	84.53%	77.98%
		Ours	91.96%	82.46%	86.95%	80.55%

**Table 6:** Comparison with representative PWD detection methods on the self-built cross-platform dataset.

Network	Precision	Recall	F1-Score	mAP_50_
SLMW-Net	69.84%	72.57%	71.18%	65.32%
TVIM	70.61%	71.82%	71.21%	64.89%
PWD-YOLO	68.53%	70.15%	69.33%	64.81%
YOLOv11-OC	67.93%	70.67%	69.27%	63.96%
Pine-Yolo	69.19%	71.62%	70.38%	65.11%
Ours	72.64%	74.53%	73.57%	67.78%

## Data Availability

Our self-built dataset can be accessed via the following link: https://github.com/heshicong88/Datas (accessed on 1 June 2026). The PDT Public Dataset can be accessed via the following link: https://www.selectdataset.com/dataset/c93398bde5b421bdee2f2c7dbe99604e (accessed on 1 June 2026). The Roboflow Pine Wilt Disease Public Dataset can be accessed via the https://universe.roboflow.com/project-dkq3q/-9pmdt (accessed on 1 June 2026) webpage. The trained model weights, lightweight testing code, and a subset of sample data for evaluation and review are publicly available at https://github.com/heshicong88/SPCA-Net (accessed on 1 June 2026).
